# A Discrete Class of Intergenic DNA Dictates Meiotic DNA Break Hotspots in Fission Yeast

**DOI:** 10.1371/journal.pgen.0030141

**Published:** 2007-08-24

**Authors:** Gareth A Cromie, Randy W Hyppa, Hugh P Cam, Joseph A Farah, Shiv I. S Grewal, Gerald R Smith

**Affiliations:** 1 Division of Basic Sciences, Fred Hutchinson Cancer Research Center, Seattle, Washington, United States of America; 2 Laboratory of Molecular Cell Biology, National Cancer Institute, National Institutes of Health, Bethesda, Maryland, United States of America; Brandeis University, United States of America

## Abstract

Meiotic recombination is initiated by DNA double-strand breaks (DSBs) made by Spo11 (Rec12 in fission yeast), which becomes covalently linked to the DSB ends. Like recombination events, DSBs occur at hotspots in the genome, but the genetic factors responsible for most hotspots have remained elusive. Here we describe in fission yeast the genome-wide distribution of meiosis-specific Rec12-DNA linkages, which closely parallel DSBs measured by conventional Southern blot hybridization. Prominent DSB hotspots are located ∼65 kb apart, separated by intervals with little or no detectable breakage. Most hotspots lie within exceptionally large intergenic regions. Thus, the chromosomal architecture responsible for hotspots in fission yeast is markedly different from that of budding yeast, in which DSB hotspots are much more closely spaced and, in many regions of the genome, occur at each promoter. Our analysis in fission yeast reveals a clearly identifiable chromosomal feature that can predict the majority of recombination hotspots across a whole genome and provides a basis for searching for the chromosomal features that dictate hotspots of meiotic recombination in other organisms, including humans.

## Author Summary

Homologous genetic recombination has two immediate benefits for cells—faithfully repairing broken DNA and aiding chromosome segregation during the first division of meiosis. Meiosis comprises a pair of special nuclear divisions that convert diploid somatic cells into haploid sex cells; in humans, meiosis leads to formation of eggs and sperm. By introducing double-strand breaks (DSBs) into their own DNA during meiosis, organisms promote recombination and hence production of viable sex cells. Although meiotic DSBs, and therefore recombination, occur throughout genomes, they arise at high frequency in certain genomic regions called hotspots, whose molecular bases are rarely understood. In this article we determine the locations of DSBs across the entire genome of the fission yeast Schizosaccharomyces pombe by taking advantage of physical linkages between DNA and the protein Rec12 that makes DSBs. This analysis shows that most of the DSB hotspots are in exceptionally large intergenic (gene-free) regions spaced on average about 65 kb apart and making up only a small fraction of the genome. Between the hotspots we see very little evidence of DSBs. The concentration of hotspots in large intergenic regions suggests that DSBs may be determined by special nucleotide sequences buried in these regions. Determining these special sequences will allow predictions of hotspots and, perhaps, the proteins and features of genome architecture that lead to DSBs being made at these special sites.

## Introduction

Genome-wide analysis of a molecular event can provide insights, such as identification of patterns within the data at multiple scales, which are unavailable through study of single events. Using the fission yeast *Schizosaccharomyces pombe,* we analyze an event, the formation of DNA double-strand breaks (DSBs), which is essential for meiotic recombination. We find that hotspots of DSB formation occur primarily in unusually large intergenic regions (IGRs) and are widely separated by apparently break-free regions. Our results show that a clearly identifiable subset of noncoding DNA has a special function that is critical for meiosis. These results contrast with those obtained in the distantly related budding yeast *Saccharomyces cerevisiae.*

Meiosis consists of two special nuclear divisions during which chromosome number is reduced from two in somatic cells to one in gametes. Uniquely, in the first meiotic division homologs, rather than sister chromatids, segregate from each other. In most organisms homolog segregation is aided by crossover recombination.

Meiotic recombination is initiated by DSBs in both budding and fission yeasts and perhaps in all species [1–3]. Repair of the DSBs by interaction with a homolog produces the crossovers (reciprocal recombination events) important for homolog segregation and for the generation of genetic diversity. DSBs are formed by a meiosis-specific protein Spo11 (Rec12 in S. pombe) through a topoisomerase-like mechanism, in which a tyrosine residue of the protein forms a phosphodiester covalent linkage with each 5′ end at the DSB [3,4]. DSBs can be detected by extraction of DNA from meiotic cells and analyzing it by Southern blot hybridizations, which reveal distinct hotspots of break formation. DSBs are transient in wild-type cells but accumulate to high levels in mutants, such as *rad50S,* in which the Spo11 or Rec12 protein is not removed from the ends of breaks [2,5]. Analysis of DSBs is thus more sensitive in *rad50S* cells.

DSB hotspots were first detected at recombination hotspots, sites at which gene conversion (nonreciprocal recombination) occurs at especially high frequency. Gene conversion hotspots have been identified in the wild-type chromosomes of many species, including humans, and may be a universal aspect of meiotic recombination. A hotspot created by the *ade6-M26* mutation in S. pombe has been especially informative. The single bp mutation *M26* creates a binding site for the transcription factor Atf1-Pcr1, which is essential for hotspot activity and DSB formation at *M26* [6–9]. Although a few meiotic hotspots in S. pombe are binding sites for this transcription factor [9,24], most hotspots are not, since only a few DSBs depend on Atf1-Pcr1. Similarly, in S. cerevisiae a few hotspots depend on the Bas1 transcription factor [10]. The determinants for the majority of the hotspots have been unclear.

To determine the global distribution of DSBs in *S. pombe,* we have used two methods—an extensive analysis of DSBs by Southern blot hybridizations and a genome-wide microarray analysis of Rec12-DNA linkages. We find that these two methods agree remarkably well and show, surprisingly, that the small class of exceptionally large IGRs is highly predictive and contains the majority of meiotic DSB hotspots.

## Results

### Evidence for Meiosis-Specific Covalent Linkage of Rec12 to Hotspot DNA

Previous evidence suggested that Rec12, like S. cerevisiae Spo11, becomes covalently linked to DNA during meiosis. Rec12 Tyr^98^ corresponds to a Tyr residue present in all analyzed Spo11 proteins [4], and the alteration Tyr^98^ → Phe^98^ abolishes meiotic recombination [3]. The corresponding Tyr^135^ of S. cerevisiae Spo11 is essential for recombination and DSB formation and is thought to be the active site residue linked to DNA [4]. To confirm Rec12-DNA covalent linkage, we analyzed, by locus-specific PCR, DNA in immunoprecipitates (IPs) of chromatin from *rec12-FLAG rad50S* cells without an exogenous crosslinking agent; the Rec12-FLAG protein is fully active in meiotic recombination (Table S1). We analyzed DNA at two known hotspots—the *M26*-related *ade6–3049* mutational hyper-hotspot [11] and the natural *mbs1* hotspot [12]. We also analyzed the *ura1* locus, at which DSBs are not detectable by Southern blot hybridization [5]. At both hotspots, but not at *ura1*, strong PCR signals were observed at 4, 5, and 6 h after meiotic induction (Figure S1), in parallel with the accumulation of DSBs at these times in *rad50S* cells as measured by Southern blot hybridizations [5]. This meiosis-specific signal was dependent on the FLAG antibody (unpublished data), the FLAG tag on Rec12, and intracellular Rec10 protein, which is also essential for recombination and DSB formation [13]. Approximately 30 times more signal was observed with primers specific to *ade6,* which contains a DSB hotspot, than with primers specific to *ura1,* which lacks detectable DSBs. As expected, approximately equal yields of product using the whole cell extract (WCE) (nonimmunoprecipitated) DNA were obtained at the *ade6–3049, mbs1,* and *ura1* sites. Since no crosslinking agent such as formaldehyde was used, we infer that the observed PCR signals using the IPs reflect covalent linkage or very tight binding of Rec12 specifically to DNA at meiotic hotspots.

### Genome-Wide Analysis of Rec12-DNA Linkages

To extend this analysis to the entire genome, we analyzed DNA in IPs of chromatin prepared from cells harvested before and 5 h after meiotic induction, using tiling microarrays of oligonucleotides (60-mers; “probes”) spaced on average every 300 bp across the S. pombe genome [14]. These arrays have been used to analyze the genome-wide distribution of heterochromatin proteins and histone modifications [14]. We have analyzed three meiotic inductions, two with a haploid strain (Dataset S1) and one with a diploid (Dataset S2). These strains are *pat1–114* (Ts) mutants, which initiate meiosis synchronously when the temperature is raised, even from the haploid state [15]; where tested, haploids and diploids give DSB patterns indistinguishable by Southern blot analysis [5].

Using the tiling microarrays, we compared the Rec12-DNA signals obtained using the IPs to those obtained using WCEs. The ratio of the IP:WCE signals at each probe reflects the enrichment of DNA linked to Rec12. This ratio was normalized using the median values of the IP and WCE signals for all of the ∼41,000 probes [14]. Because the chromatin DNA was sheared to ∼0.5–1 kb before immunoprecipitation, and because Spo11 (and likely Rec12) becomes linked to both 5′ ends at a DSB [4], we expect genuine Rec12-DNA signals to hybridize to at least three adjacent probes on the array. For our subsequent analysis we thus excluded isolated single or double probes (“spikes”) with high values (IP:WCE ratios >2), as done similarly by Cam et al*.* [14]. High points in the induced (5 h) data corresponding to spuriously high uninduced (0 h) points were also removed. The effect of this filtering and its comparison with other noise-reducing methods are shown in Figure S2. High Rec12 enrichment ratios in the 0-h data are restricted to isolated spikes, whereas in the 5-h data high enrichment ratios are also seen in contiguous groups of high ratio probes, indicative of genuine Rec12 enrichment (Figure S10). Filtering removed the spikes (Figure S11). As expected [16], the filtered data from the 0-h samples were approximately normally distributed, whereas the data from the 5-h samples were approximately normally distributed but with an additional, substantial non-normal distribution of high values (i.e., sites of Rec12-DNA linkage) (Figures S3 and S4). Greater variance in the diploid 0-h background distribution meant that filtering of spikes was somewhat less effective in that dataset than in the others.

**Figure 1:**
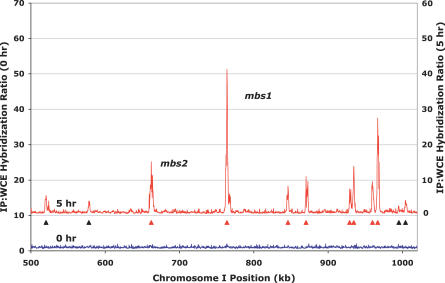
Rec12-DNA Linkages Occur at Strong Hotspots Separated by Cold Regions across *Not*I Fragment J (524-1025 kb from the Left End of Chromosome I) Haploid strain GP6013 bearing the *rec12–201::6His-2FLAG* allele was induced and analyzed for Rec12-DNA linkage by microarray analysis before (0 h; blue line) and 5 h (red line; mean of two experiments) after meiotic induction. Data are the median-normalized IP:WCE ratios for each oligonucleotide, spaced ∼300 bp apart [14]. Probes with ratios >2 were replaced with values of 1 unless they occurred in contiguous groups of >2 spots (see text for justification); high values within contiguous groups (hotspot peaks) in the 5-h data were omitted if they were also spuriously high in the 0-h data. See Figure S2 for alternative data presentations, Figure S5 for repeat experiments, and Figure S7 for the entire genome. Carets indicate weak (black) and prominent (red) Rec12 peaks identified by PeakFinder. Left and right vertical axes are offset to allow separation of the 0- and 5-h datasets.

We first focused our attention on a 0.5-Mb region near the left end of Chromosome I, defined by *Not*I restriction fragment J, which we have previously analyzed extensively for meiotic recombination and DSBs [5]. Our microarray analysis reveals a consistent distribution of Rec12-DNA linkages that closely parallels the distribution of DSBs determined by Southern blot hybridization (see next section). Strong Rec12-DNA signals (IP:WCE ratios >2 and up to ∼40) were observed at ∼20 consecutive probes at the position of the previously mapped *mbs1* and *mbs2* DSB hotspots of *Not*I fragment J in each of the three inductions (5-h data; Figures 1 and S5). Additional strong hotspots of Rec12-DNA linkage were observed at six other sites to the right of the *mbs2–mbs1* pair. This pattern was consistent for all three inductions, and the hotspots were essentially absent in the uninduced (0-h) samples (Figure S5). Between the obvious hotspots in the 5-h DNA the signals varied in a small range around a ratio of 1, similar to the 0-h data and consistent with the expected background (non-Rec12 enriched) distribution [16]. Thus, the microarray analysis reveals strong hotspots of Rec12-DNA linkage separated by large regions in which linkage is undetectable (see additional discussion below).

### Rec12-DNA Linkage Analysis by Microarrays Closely Parallels Direct DSB Analysis by Southern Blot Hybridization

**Figure 2:**
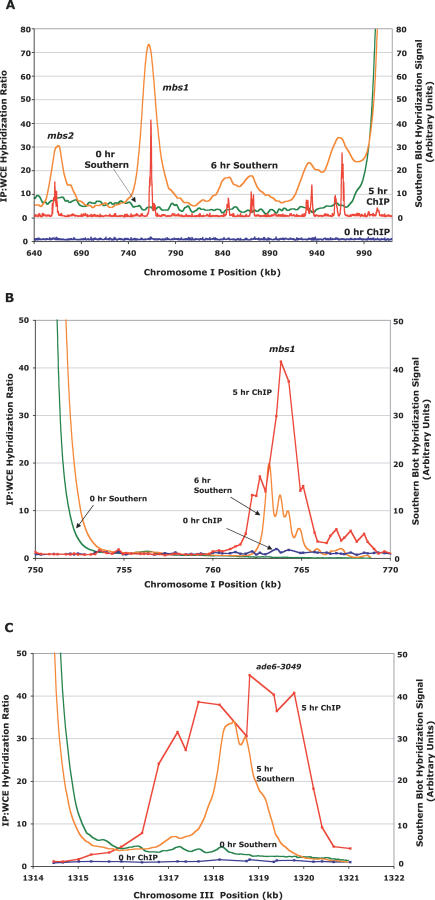
Rec12-DNA Linkages Strongly Correlate with DSBs Determined by Southern Blot Hybridization Haploid microarray data as in Figure 1 are plotted with tracings of a Southern blot of DNA that was prepared during a meiotic induction at the indicated times, subjected to gel electrophoresis, blotted, probed, and analyzed by Phosphorimager. (A) *Not*I-digested DNA probed from the left end of 501-kb fragment J [5]. (B) *Mlu*I-digested DNA probed from the right end of the 20.9-kb fragment with *mbs1*. (C) *Afl*II-digested DNA probed from the right end of the 6.6-kb fragment with *ade6* [9].

We directly compared the distribution of Rec12-DNA linkages to that of DSBs determined by Southern blot analysis. A close correlation of both positions and intensities was seen across *Not*I fragment J (Figure 2A). At this scale (50–500-kb DNA fragments), the microarray data provide higher resolution and a higher signal-to-noise ratio than Southern blot analysis. Comparison of the microarray data with direct DSB analysis of shorter DNA fragments gave a slightly different picture. The positions of the peaks at two well-studied DSB hotspots, *mbs1* on Chromosome I and *ade6–3049* on Chromosome III, again were coincident, but the Southern blot analysis of shorter DNA fragments now provides higher resolution than the microarray data (Figure 2B and 2C), as expected from the length of DNA linked to Rec12 (see above).

**Figure 3:**
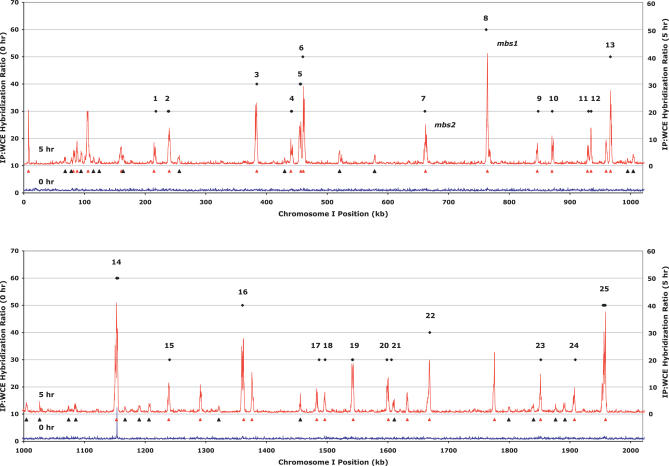
Close Correlation between DSBs and Rec12-DNA Linkages across 1.8 Mb of Chromosome I Microarray data from 0 h (blue line) and 5 h (red line) analyses of haploids are presented as in Figure 1. Numbered black diamonds indicate discernable DSB hotspots seen on Southern blots of DNA digested with a variety of enzymes that produce 10–100-kb fragments (see examples in Figures 2 and S6 for the Southern blots analyzed). Intensity and position data for *mbs1* and *mbs2* (numbers 7 and 8) are from [5,12]. Carets indicate weak (black) and prominent (red) Rec12 peaks identified by PeakFinder. The peak at 1,776 kb is at the *rec12* gene and likely results from the *rec12-FLAG* allele used for microarray analysis; no DSB hotspot was evident by Southern blot analysis of *rec12^+^* strains (Figure S7).

We extended this dual analysis beyond the DSB hotspots on *Not*I fragment J noted by Young et al*.* [5]. We first analyzed DSBs by Southern blot analysis of the *Not*I fragments adjacent to fragment J (Figure S6). This analysis encompassed ∼1.8 Mb of the left arm of Chromosome I, including *Not*I fragment J, and represented ∼15% of the S. pombe genome. We found 25 discrete DSB hotspots, or tight clusters of hotspots, scattered across the region. These hotspots were then mapped more precisely by analysis of smaller restriction fragments, ∼10–100 kb long, covering much but not all of the 1.8 Mb region. Every DSB hotspot was coincident with a hotspot of Rec12-DNA linkage independently obtained by microarray analysis (Figure 3). We did not survey at higher resolution all of the weak Rec12-DNA linkage peaks or stronger DSB sites whose diagnostic DNA fragments were poorly separated from those of nearby DSB sites. Except for these few Rec12-DNA linkage sites that have not been tested by Southern blot analysis, there is a strict correspondence of Rec12-DNA linkage and DSB hotspots.

### Signal Strengths by the Two Measures Are Similar

**Figure 4:**
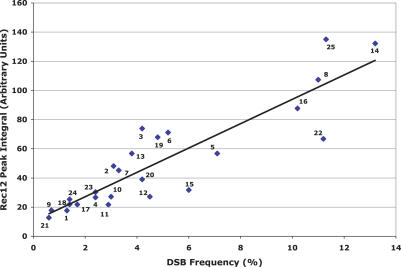
Hotspot Strengths Measured by Southern Blots of DSBs and by Microarray Data of Rec12-DNA Linkages Correlate Well The percent of total DNA broken at each of the 25 hotspots indicated by numbered black diamonds in Figure 3 is plotted versus the integral of the IP:WCE ratios for the corresponding peaks in the microarray data (mean of haploid and diploid experiments). The regression line is *y =* 8.35*x* + 10.4.

We noted above that the relative signal strengths of the peaks on the 0.5 Mb *Not*I fragment J were similar by Rec12-DNA and direct DSB analyses (Figure 2A). We extended this comparison for the 25 peaks seen by both measures in the 1.8 Mb region shown in Figure 3. We observed a strong trend between the strength of the DSB signals (percent of DNA broken at the site) and the strength of the microarray signals (IP:WCE ratios integrated over the peak) (Figure 4). The Pearson correlation coefficient *r* was 0.89 (*p* < 0.001 by t-test; *r* ^2^ = 0.79); the Spearman rank correlation coefficient ρ was 0.90 (*p* < 0.001 by t-test).

The data in Figures 2–4 show that the microarray data faithfully reflect direct assay of DSBs by Southern blot analysis, with respect to both the position of the DSBs and the amount of DNA breakage (strength of the hotspot). This outcome allows us to use the microarray data as a reliable substitute assay for DSBs across the entire genome. Hereafter, we use “DSB hotspots” and “Rec12 peaks” interchangeably. Because the limit of detection on our Southern blots is ∼0.5% (Figure S6), we consider sites with ≥0.5% estimated DSBs, based on the relation in Figure 4, to be “prominent” DSB hotspots or Rec12 peaks and those with <0.5% to be “weak.” From the peak integrals we estimate that the prominent Rec12 peaks represent ∼90% of all detectable Rec12-DNA linkages (see below). In the Discussion, we describe additional differences between the prominent and weak hotspots, which suggest that they are distinct.

### DSB Hotspots Preferentially Occur in Large IGRs

**Figure 5:**
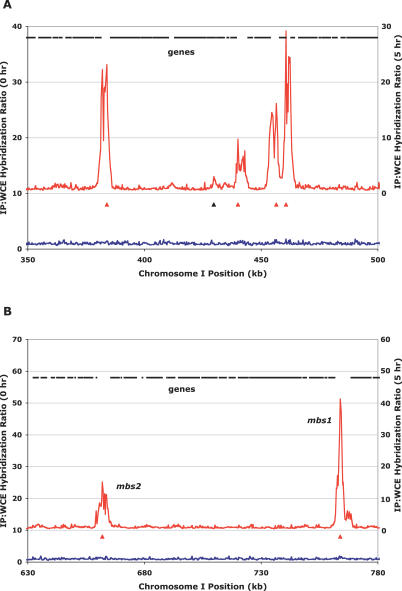
DSB Hotspots Occur in Large IGRs Microarray data for haploid 0-h DNA (blue line), the mean of two 5-h DNA analyses (red line) processed as in Figure 1, and coding sequences (genes, black lines at top) from the annotated S. pombe genome (Build 1.1; http://www.ncbi.nlm.nih.gov/entrez/query.fcgi?db=genomeprj&cmd=Retrieve&dopt=Overview&list_uids=9517) are shown for two 150-kb intervals of Chromosome I. (A) An interval to the left of that containing *mbs2–mbs1* (see Figure 1)*.* (B) The *mbs2–mbs1* region. Carets indicate weak (black) and prominent (red) Rec12 peaks identified by PeakFinder.

Young et al*.* [5] noted that the DSB hotspot *mbs1* is located in an IGR that is especially large—7.2 kb. The mode of S. pombe IGRs is 0.4 kb, and the median is 0.7 kb [17]. *mbs2* is also in a large IGR—6.3 kb. We therefore examined the microarray data for the location of hotspots and found a strong correlation between DSB hotspots and large IGRs. (For this analysis we classed as intergenic the putative coding sequences annotated as “dubious” or “very hypothetical.”) For example, in the 150-kb interval that includes *mbs1* and *mbs2* there are no other prominent hotspots and no other IGRs larger than 1.9 kb (Figure 5B). A nearby 150-kb interval on the left arm of Chromosome I has four prominent hotspots, which fall in IGRs of size 5.2, 4.3, 4.1, and 2.3 kb (Figure 5A). In these 300 kb all IGRs >2.8 kb have prominent hotspots, and five out of six such hotspots occur in IGRs >4 kb. In the 1.8-Mb region depicted in Figure 3, 16 of the 25 prominent Rec12 peaks confirmed by Southern blot analysis fall in IGRs >2.8 kb, five fall in IGRs of 0.6–2.3 kb, and the remaining four fall in coding sequences.

We extended this analysis genome-wide, except for the telomeres and centromeres, which are considered separately in the Discussion. Rec12-DNA linkage positions were identified as the maxima of Rec12 peaks using the PeakFinder program [18]. Using the mean of the haploid 5-h data, this program identified 353 such positions genome-wide. The 149, 124, and 80 positions seen on Chromosomes I, II, and III respectively (Figure S7) are in good agreement with the relative sizes of the chromosomes (5.6, 4.5, and 2.5 Mb, not including the rDNA at the ends of Chromosome III). Of the 353 Rec12 peak positions from the haploid data, 340 (96%) were also identified in the diploid data. Using a cutoff of *p* < 10^−8^, a cutoff that excludes all but 18 (12%) of the false-positive peaks identified in the 0-h data, the ChIPOTle program [19] independently identified peak regions in the haploid data that included all of the positions identified by PeakFinder, plus an additional 16 (4%). We used the peak positions identified by PeakFinder from the haploid data for the analysis below.

As mentioned above, we estimated the relative frequency of Rec12-DNA linkage at each hotspot by integrating the IP:WCE ratios across each peak and classified the peaks as “prominent” (≥0.5% estimated DSBs) or “weak” (<0.5%) based on the relation in Figure 4. Among the 353 peaks identified by PeakFinder, 194 are prominent hotspots. Based on the sum of the integrals, these prominent peaks account for 88% of all detectable Rec12-DNA linkages (DSBs).

**Figure 6:**
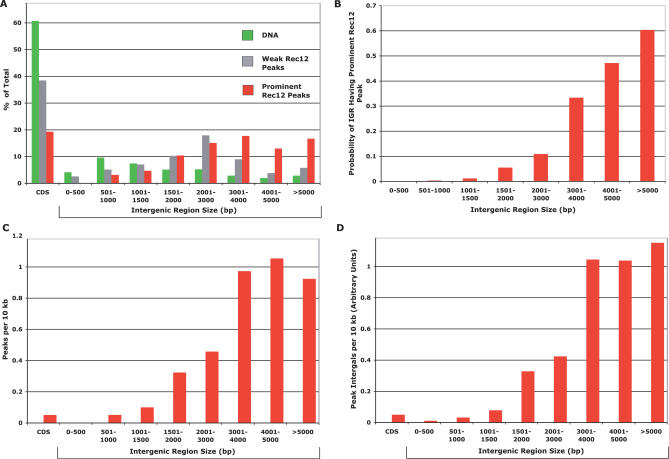
Most DSB Hotspots Occur in Large IGRs Frequency distributions of DNA (green bars), weak Rec12-DNA linkage sites (gray bars), and prominent Rec12-DNA linkage sites (red bars) are shown for the classes of DNA sequence indicated. Analysis was carried out using the whole genome excluding the centromeres and 50 kb at the end of each chromosomal contig (to exclude telomeric regions). (A) Number distribution. Peaks of Rec12-DNA linkage (DSB hotspots) identified from the mean of the haploid 5-h data were classified according to their position in coding sequences (CDS) or in IGRs. A total of 62% of prominent Rec12 peaks occur in IGRs >2 kb. (B) Probability of a prominent Rec12 peak occurring within an IGR increases with IGR size. (C) Density of prominent Rec12 peaks (number of peaks per kb) increases with IGR size. (D) DSB density (Rec12 peak integrals per kb) is greatest in large IGRs.

Our analysis shows that DSB hotspots are over-represented in large IGRs and under-represented in coding DNA. Figure 6A shows the proportion of all genomic DNA and the proportion of all prominent and weak Rec12 peaks found in coding sequences and in IGRs of different sizes. The distributions of all DNA and Rec12 peaks are clearly distinct (for prominent peaks *p* < 0.0001 and for weak peaks *p* < 0.005; two-tailed contingency chi-squared test). IGRs >2 kb comprise only 13% of the genome, but they contain 62% of all prominent Rec12 peaks and 37% of the weak Rec12 peaks. Similarly, IGRs >3 kb comprise only 8% of the genome but contain 47% of the prominent Rec12 peaks and 19% of the weak Rec12 peaks. Thus, prominent peaks are more strongly enriched in large IGRs than are weak peaks. In fact, among prominent peaks, the probability of a peak lying in an IGR >3 kb rises with peak integral so that the very strongest peaks are even more highly associated with large IGRs (Figure S8). The Rec12 peak integrals, a measure of Rec12-DNA linkages and hence DSBs, are similarly skewed toward large IGRs: 65% of all Rec12-DNA linkages are in IGRs >2 kb, and 52% are in IGRs >3 kb. In addition, fewer prominent hotspots than weak hotspots (19% versus 38%) occur in coding DNA, which accounts for 61% of the genome.

Prominent DSB hotspots, as indicated by sites of Rec12-DNA linkage, are therefore particularly strongly associated with large IGRs. In addition, large IGRs are highly predictive of DSB hotspots, since 44% of the IGRs >3 kb long have prominent Rec12 peaks (Figure 6B) and an additional 12% have weak Rec12 peaks. Furthermore, prominent Rec12 peaks are about 20 times more dense (peaks per kb) in IGRs >3 kb than in coding sequences or short IGRs (Figure 6C). The density of DSBs (peak integrals per kb) is also 20–40 times greater in IGRs >3 kb than in coding sequences or in short IGRs (Figure 6D). We discuss below the significance of these observations.

## Discussion

A current critical question is which genomic features and underlying nucleotide sequences distinguish meiotic DSB hotspots from other portions of the genome. Our global microarray analysis permits a genome-wide analysis of meiotic DSBs in fission yeast. The distribution of meiotic DNA covalently linked to Rec12 is nearly identical to the distribution of meiotic DSBs determined directly by Southern blot hybridizations. This correspondence holds both for the presence of DSB hotspots (Figures 2 and 3) and for their relative strengths (Figure 4). We can thus use the more easily determined Rec12-DNA linkage data from microarray analysis as a valid proxy for DSBs genome-wide. Because the limit of detection on our Southern blots is ∼0.5% (Figure S6) [5], we concentrate on prominent Rec12 peaks, those whose integrals indicate a DSB frequency ≥0.5% (Figure 4). Our analysis of the 194 prominent hotspots that we identified in the 12.6-Mb S. pombe genome shows that prominent DSBs are detectable at hotspots spaced on average ∼65 kb apart (Figure S7). Between these hotspots there is very little, if any, Rec12-DNA linkage detectable (Figures 1–3 and 5) or DSBs detectable by Southern blot analysis (Figure 2A) (unpublished data) [5]. We estimate from our microarray data that DSBs collectively occur in ∼700 kb of DNA, or ∼6% of the genome. Below, we discuss current knowledge of what is special about this small portion of the genome.

Most analyses of meiotic DSBs in S. pombe and S. cerevisiae have used the *rad50S* (K81I) mutation to allow accumulation of DSBs and thus a higher DSB level at most hotspots [20,21]. In one analysis using both *RAD50* and *rad50S S. cerevisiae,* DSBs were reduced in some but not other intervals in *rad50S* relative to *RAD50* [22]. In S. pombe the number, position, and relative intensities of DSB hotspots are not significantly different in *rad50S* and *rad50^+^* strains (Figure S9). This difference in the two yeasts may reflect Rad50 being required for DSB formation in S. cerevisiae but not in S. pombe [2,23]. Furthermore, the *pat1–114* mutation, which allows a more nearly synchronous induction of meiosis than that in *pat1^+^* strains, has little if any effect on the DSB pattern (Figure S9). Thus, the analyses presented here appear to reflect the DSB pattern in wild type.

Young et al. [5] analyzed extensively the 0.5-Mb *Not*I fragment J represented in Figure 1 and noted a noncongruence between the distribution of DSBs and crossovers. Our microarray data confirm this noncongruence, which remains an unsolved paradox.

The only known determinant of the position of meiotic DSB hotspots in S. pombe is the transcription factor Atf1-Pcr1 and its DNA binding site [24 and references therein]. Of the 15 genomic sequences closest to the consensus sequence for binding of Atf1-Pcr1 to naked DNA, 11 are DSB hotspots demonstrable by Southern blot hybridization [24], and we identified ten of these by microarray analysis (Figure S7) (unpublished data). We note, however, that the consensus sequence is not sufficient for DSB formation: four loci with this sequence do not have DSBs detectable by either method, and most *ade6-M26* transplaced fragments with an Atf1-Pcr1 binding site are not recombination hotspots [25,26]. Nor is Atf-Pcr1 binding required for the DSB formation at most hotspots [24]. Nevertheless, the ability to successfully predict DSB hotspots from the Atf1-Pcr1 consensus sequence encourages the search for other sequences or genomic features that determine hotspots.

### Meiotic DSBs Are Concentrated in Large IGRs

The most prominent feature associated with DSB hotspots that we have noted is exceptionally large IGRs (Figures 5 and 6). Approximately 50% of the IGRs >3 kb in length have Rec12 peaks (Figure 6A), which collectively account for approximately 50% of all detectable Rec12-DNA linkages (DSBs). Thus, this genomic feature is highly predictive. We presume that one or more nucleotide sequences, currently unknown but restricted to the IGRs with DSBs, are essential for DSB formation. These sequences might be collections of transcription factor binding sites, exemplified by *M26,* the binding site for Atf1-Pcr1. The S. cerevisiae transcription factor Bas1 and its binding site also account for a small fraction of the meiotic DSB hotspots observed in that distantly related yeast [10]. We think it likely that transcription factors other than Atf1-Pcr1 also influence DSB formation in S. pombe. Transcription factor binding sites are concentrated in IGRs and collectively may account for a significant fraction of meiotic DSBs. Other sequences, such as those affecting nucleosome positioning, may determine additional DSB hotspots. The data reported here provide a framework for identifying such determinants.

Ludin and colleagues also noted an enrichment of Rec12 in IGRs in their genome-wide analysis of Rec12 crosslinked with formaldehyde to meiotic DNA (K. Ludin, J. Mata, J. Bahler, and J. Kohli, personal communication). In their analysis Rec12 was more uniformly distributed across the genome than in our analysis, perhaps a reflection of only a fraction of chromatin-bound Rec12 being active in making DSBs and becoming crosslinked without an exogenous agent.

We observed that DSB hotspots are over-represented in IGRs whose flanking genes are divergently transcribed, compared to their frequency in all IGRs (*p* < 0.001; contingency chi-squared test). However, the orientation of transcription of adjacent genes is itself correlated with the size of the IGR [17], and no enrichment for DSB hotspots was seen in divergent IGRs when the size of the IGR was controlled for (unpublished data). We know of no clear evidence that transcription *per se* influences meiotic DSB formation (e.g., [8]).

### Relation of Meiotic DSBs and Other Genomic Features

We searched for other features of the S. pombe genome that might correlate with DSB hotspots and thus be important factors for their determination. The meiosis-specific cohesin subunit Rec8 is required for most DSB formation in S. pombe [13]. Rec8 is thought to be part of the chromosomal core that forms the basis of the axial elements (linear elements in S. pombe) and from which chromatin loops emanate [27]. Studies in S. cerevisiae have led to the conclusion that DSB hotspots preferentially occur in the loops, where Rec8 density is low [18]. S. pombe Rec8 is distributed nonuniformly, at least over the portion of the genome surveyed (Chromosome II and part of Chromosome III) [28]. We observed a weak negative correlation between Rec8 binding and Rec12-DNA linkage. For example, 83% of the 69 prominent Rec12 peaks on Chromosome II are centered on an 11-probe window whose Rec8-binding ratio is below the median for Chromosome II (ignoring the centromere), significantly different from the 50% expected (*p* < 0.001; contingency chi-squared test). Among the 55 weak Rec12 peaks, this bias falls to 64%, not significantly different from 50%. Conversely, the quarter of probes on Chromosome II with the highest Rec8 enrichment ratios contain only 9% of the prominent Rec12 peaks, less than the 25% expected, and the quarter with the lowest Rec8 enrichment ratios contain 55% of the prominent Rec12 peaks, more than the 25% expected. Therefore, in S. pombe as in S. cerevisiae, DSBs are associated with regions of low Rec8 localization, but neither distribution is very useful for predicting the other.

As noted above, Atf1-Pcr1 and perhaps other transcription factors are required for some DSB hotspots. We therefore looked for a correlation between DSB hotspot positions and the promoters of genes induced during meiosis. Among such genes [29], we found no significant enrichment of Rec12 peaks 5′ of the corresponding open reading frame. We then looked among all genes with Rec12-peaks in their presumptive promoters for enrichment of related functions, as indicated by related gene ontology (GO) terms. Using the GOstat Web server (http://gostat.wehi.edu.au) [30] with Benjamini correction for multiple hypothesis testing, we found a significant enrichment for terms, such as GO:0051704 (Interaction between organisms), GO:0000746 and GO:0019953 (Conjugation and Sexual reproduction, subsets of GO:0051704), and GO:0003700 (Transcription factor activity), listed in Table S2. The causal basis of these correlations is not obvious. However, the enrichment of Rec12 peaks in the promoter regions of genes associated with conditions and processes related to meiosis and meiotic induction suggests that meiosis-specific chromatin remodeling may occur at some promoters, allowing access or activation of Rec12 for DSB formation. A particular group of transcription factors and transcription factor-binding sequences may be involved. As noted above, this is the case for the *M26* hotspot and Atf1-Pcr1, factors involved in the meiosis-specific remodeling of chromatin and Rec12-dependent DSB formation [31–33].

In S. pombe mitotic replication origins are spaced about 25–30 kb apart, and, where tested, the same origins are used during both mitotic growth and meiosis [34]. We found, however, no obvious relationship between origins of replication and meiotic DSB hotspots. For example, 14% of all replication origins correspond to haploid 5-h probe positions with a Rec12 IP:WCE ratio >2 (i.e., all regions of Rec12-DNA linkages), not significantly different from the number expected based on the proportion (11%) of all probes with a Rec12 enrichment value >2 (*p* > 0.05; contingency chi-squared test). Therefore, the frequency of origins of replication is neither enriched nor depleted significantly in regions of Rec12-DNA linkage.

We looked for an unusual nucleotide sequence composition surrounding DSB hotspots and found only a weak correlation with higher than average guanine-cytosine (GC) content. For example, 5-kb windows centered on the 194 prominent Rec12 peak positions have a slightly enriched GC content compared to 5-kb windows, moved in 100-bp steps, covering the whole genome (median of 36.6% versus 36.0%; *p* < 0.0001 by two-tailed t-test). However, base composition is a very poor predictor of prominent Rec12 peak position, as 37% of prominent Rec12 peaks are centered on windows with GC content below the global median and only 0.2% of windows with GC content above the global median are centered on a prominent Rec12 peak. No significant GC content enrichment was seen at weak Rec12 peak positions. Sliding windows of 0.5 kb, 1 kb, or 10 kb showed even less difference between peak-centered and whole-genome window compositions (unpublished data). In *S. cerevisiae,* DSB hotspots are also weakly correlated with higher than average GC content when a 5-kb sliding window is used [20].

### Comparison of *S. pombe, S. cerevisiae,* and Human DSB Hotspot Distributions

As in *S. pombe,* DSBs have been analyzed in S. cerevisiae by both direct Southern blot analysis and microarray analysis of DNA covalently linked to Spo11, the Rec12 homolog [e.g.,20,21,35]. These analyses show that DSB hotspots in S. cerevisiae are in general slightly less prominent and often more closely spaced than those in S. pombe. For example, on the most thoroughly analyzed S. cerevisiae Chromosome III, the maximal fraction of DNA broken at a hotspot is 9% [35], and the maximal enrichment of Spo11-DNA linkage is 8-fold [20] or 15-fold [21]. In many regions of the genome, most of the open reading frames have a detectable DSB at their 5′ end.

In S. pombe many DSB hotspots have 10%–15% of the DNA broken (Figures 4 and S6) [5], and Rec12-enrichment factors (IP:WCE ratio) are often >25 and occasionally up to 50 (Figures 3 and S7). Between the prominent Rec12 peaks, there are frequent intervals of 50–100 kb with few if any DSBs detectable by either Southern blot or microarray analysis (Figures 3 and S7) [5]. We have not noted continuous stretches of genes with DSB hotspots at their 5′ ends. Thus, DSB hotspots (or small groups of hotspots) are usually isolated from others in *S. pombe.* In spite of hotspots being far apart, crossovers in S. pombe are nearly uniformly distributed [5]. Crossing-over far from detectable DSBs may involve migration of DNA intermediates far from a DSB, or they may be initiated by DNA lesions not detectable by current technology [12,36].

In these two yeasts meiotic DSBs are distributed differently in the centromeric and telomeric regions than in other regions of the genome. In S. cerevisiae centromeres are small (∼0.1 kb), and DSBs are infrequent to one or both sides of many of the centromeres [10,20,21,35]. In S. pombe centromeres are large (35–100 kb), and DSBs are undetectable within them by microarray analysis (Figure S7) or by Southern blot analysis (C. Ellermeier and G. Smith, unpublished data). In the adjacent intervals, however, DSBs appear as frequent as in other regions of the genome. In both yeasts ChIP-on-chip analysis suggests that Rec12 (Spo11)-DNA linkages are under-represented in the terminal 40 kb by ∼20% (unpublished data) [10,20,21]. In *S. pombe,* however, there is a prominent DSB ∼18 kb from the left end of Chromosome I, including ∼10 kb of unsequenced DNA (Figure 3), although prominent Rec12 peaks occur only 70–150 kb from the other three telomeres. DSB-free intervals of similar size occur throughout the chromosomes. Thus, with respect to meiotic DSBs S. pombe telomeres do not appear substantially different from other regions of the genome.

Meiotic DSBs have not been directly detected in human cells, but hotspots of recombination, presumably reflecting hotspots of DSB formation, have been detected using both linkage disequilibrium analysis and sperm typing. These methods have detected small regions of DNA, often only 1–2 kb wide by sperm typing, where meiotic recombination rates are greatly elevated compared to neighboring intervals or the genome average [37–40]. Like S. pombe and *S. cerevisiae,* human DSB formation and recombination appear to be dominated by hotspots accounting for only a small proportion of the total genome and located most frequently in IGRs of the genome [40,41]. Human hotspots are located 50–100 kb apart [39,42], similar to the distribution of hotspots in S. pombe (Figure 3). The strongest human hotspot has a crossover frequency of ∼1% [43], lower than that at *mbs1* (5%) [12] and presumably than at several even stronger DSB hotspots in S. pombe (Figures 3 and S7). Human DSB hotspots are expected to be weaker than those of *S. pombe,* given the similar hotspot spacing and map sizes of the two organisms but greatly larger physical genome size of humans. Like S. pombe and *S. cerevisiae,* human hotpots are positively, but only weakly, correlated with GC content; this correlation has low predictive power [44]. Therefore, several characteristics of meiotic DSB hotspots are common to *S. pombe, S. cerevisiae,* and humans, but there are also distinct differences in any pair-wise comparison of the organisms.

Further comparisons of meiotic DSB patterns in these yeasts and additional species will help understand how this process, crucial to sexual reproduction, is controlled. The analysis reported here provides a foundation for identifying the genomic features that regulate meiotic DSB formation, a problem so far unsolved for any organism.

## Materials and Methods

### S. pombe strains.

The genotypes of strains and the sources of published alleles are in Table S3. The *rec12–201::6His-2FLAG* allele, called *rec12-FLAG* in the text, was constructed as plasmid pJF32 (Text S1). The 1.6-kb *Bam*HI fragment of pJF32 was used to transform strain GP5622 (*rec12–171::ura4^+^*) to fluoro-orotic acid-resistance to generate strain GP5960; the chromosomal allele was confirmed by automated nucleotide sequence analysis. The *rec12–202::6His-2HA* allele, called *rec12-HA* in the text, was similarly constructed as plasmid pJF41 and transferred to the chromosome to generate strain GP5961. Strains with these alleles are recombination proficient (Table S1). Other strains were constructed by standard meiotic crosses.

### Preparation of meiotic chromatin and Rec12-FLAG covalently linked to DNA and its analysis with microarrays.

Cells were grown and induced for meiosis as described [5] and then harvested, washed, and stored at −20 °C. Chromatin was prepared, sonicated to yield DNA 0.5–1 kb long as determined by agarose gel electrophoresis (unpublished data), and subjected to immunoprecipitation with anti-FLAG antibody (Sigma, http://www.sigmaaldrich.com) as described [45]. Precipitates (IP) were washed, treated with proteinase K, extracted with phenol-CHCl_3_, and treated with RNase; the residual DNA was purified on a QIAquick column (Qiagen, http://www1.qiagen.com), amplified in the presence of amino-allyl dUTP, and labeled with Cy5 as described [14]. DNA from unfractionated, sonicated chromatin (WCE) was prepared and labeled with Cy3 in parallel. The samples were mixed and hybridized to an Agilent 44-K S. pombe oligonucleotide microarray and analyzed as described [14]. The IP:WCE ratio for each probe on the array (∼41,000) was then calculated after adjustment of the IP values to equalize the IP and WCE medians. Data were analyzed with Excel (Microsoft, http://www.microsoft.com), PeakFinder [18], and ChIPOTle [19] software. Peakfinder was run using the haploid and diploid datasets after noise reduction as in Figure 1, with linear plotting, Gaussian weighting, a single round using a 13-probe window, right and left minimum smoothed delta = 100, and a threshold of 2,000. ChIPOTle was run on the haploid dataset with noise reduction as in Figure S3. The Gaussian background setting was used with a window size of 1 kb, a step size of 150 bp, and a cutoff of *p* < 0.001.

## Supporting Information

Dataset S1 Median-Normalized Genome-Wide IP/WCE Hybridization Ratios from Two Meiotic Inductions of Haploid Strain GP6013 Shown are experiment 2 data from before induction (0 h) and 5 h after induction (5 h) and experiment 1 data from 5 h after induction (5 h).
(6.7 MB XLS)

Dataset S2 Median-Normalized Genome-Wide IP/WCE Hybridization Ratios from a Meiotic Induction of Diploid Strain GP6203 Shown are data from before induction (0 h) and 5 h after induction (5 h).
(6.0 MB XLS)

Figure S1 Meiosis-Specific Rec12-DNA Linkage Requires Rec10 and Is Abundant at the *mbs1* and *ade6–3049* DSB Hotspots but Not in the *ura1* Cold Region (A) Haploid strain GP6013 (*rec12–201::6His-2FLAG ade6–3049*) was induced for meiosis, and sonicated chromatin was prepared at the indicated time. Anti-FLAG precipitates (Rec12-ChIP) and unfractionated DNA (WCE) were analyzed by PCR with primers specific for the indicated loci (Table S4). The PCR products were analyzed by agarose gel electrophoresis and staining with ethidium bromide. Experiments were done three to five times with comparable results. In these experiments DNA replication, measured by flow-cytometry, occurred between 2 and 3 h after induction (unpublished data).
(B) Diploid strains GP6203 (*rec12–201::6His-2FLAG*) and GP6204 (*rec12–202::6His-2HA*) were induced for meiosis and analyzed as above. The relative amounts of Rec12-ChIP used for the PCR analysis with *mbs1*-specific primers are indicated.
(C) Diploid strains GP6203 and GP6233 (*rec10–175::kanMX6 rec12–201::6His-2FLAG ade6–3049*) were analyzed with the indicated primers as above. The relative amounts of WCE and Rec12-ChIP are indicated.
(D) WCE from the experiments in (C) were assayed with primers specific for the *ade6* and *ura1* loci, using the relative amounts of WCE indicated. Each panel includes size markers decreasing from 500 bp (black caret) in steps of 100 bp downward.
(648 KB PDF)

Figure S2 Comparison of Microarray Data Corresponding to the *Not*I Fragment J on Chromosome I Analyzed in Different Ways Data are the IP:WCE hybridization ratios obtained with haploid strain GP6013, before induction (blue line) and 5 h after induction (red line; mean of two experiments). Chromosome positions represent distance from the left (sequenced) end of Chromosome I.
(A) Raw data.
(B) Seven-probe sliding window. For each point the mean of the seven values centered on that point is plotted.
(C) Three-probe filter. Values >2 were replaced with values of 1 unless occurring in groups of at least three contiguous probes.
(D) Normalization using uninduced (0 h) values. For each point the 5-h value was divided by the 0-h value.
(2.8 MB PDF)

Figure S3 Quantile-Quantile Plots of IP:WCE Hybridization Ratios (log_2_) for Chromosome I from the Haploid Experiments (Strain GP6013) against Simulated Normal Values (with a Mean of 0 and Variance of 1) Uninduced (0 h) data are from haploid experiment 2; induced (5 h) data are the mean of two haploid experiments. Normally distributed IP:WCE hybridization ratios (log_2_) should result in a straight line passing through the origin.
(A) 0 h, raw data.
(B) 0 h, filtered to remove isolated (single and double) linear values >2 (i.e., log_2_ > 1), identified as in Figure S2C. Note that these filtered data are nearly normally distributed.
(C) 5 h, raw data.
(D) 5 h, data filtered using criteria from Figure S2C. Note that the 5-h data have many values clearly above the normal distribution that persist after filtering, as expected for genuine Rec12-enrichment of DNA at linkage sites. The percent of total data with an abscissa value ≥1 is indicated for each plot.
(992 KB PDF)

Figure S4 Quantile-Quantile Plots of IP:WCE Hybridization Ratios (log_2_) for Chromosome I from the Diploid Experiment (Strain GP6203) against Simulated Normal Values (with a Mean of 0 and Variance of 1) Normally distributed IP:WCE hybridization ratios (log_2_) should result in a straight line passing through the origin.
(A) 0 h, raw data.
(B) 0 h, filtered to remove isolated (single and double) linear values >2 (i.e*.*, log_2_ > 1), identified as in Figure S2C. Note that these filtered data have some values above the normal distribution.
(C) 5 h, raw data.
(D) 5 h, data filtered using criteria from Figure S2C. Note that the 5-h data have many values clearly above the normal distribution that persist after filtering, as expected for genuine Rec12-enrichment of DNA at linkage sites. The percent of total data with an abscissa value ≥1 is indicated for each plot.
(1.8 MB PDF)

Figure S5 Distribution of Rec12-DNA Linkages in Two Haploid Inductions (Strain GP6013) and One Diploid Induction (Strain GP6203) Are Similar Data for *Not*I fragment J are filtered and presented as in Figure S2C.
(1.9 MB PDF)

Figure S6 Southern Blot Analyses of DSBs in the 1.8-Mb Region at the Left End of Chromosome I (A) Physical map and Southern blot hybridizations of *Not*I fragments encompassing 0.2–2 Mb from the left end of Chromosome I probed from the right (R) or left (L), as indicated. Haploid strain GP5411 (*rad50S*) was induced, and at the times indicated DNA was extracted and analyzed for DSBs by Southern blot hybridization as described by Young et al. [5].
(B) Coarse mapping of DSB positions from Figure S6A *Not*I blots.
(C) Finer scale mapping of the DSB sites identified in Figures S6A and S2C was carried out using DNA from haploid strain GP5411 and diploid strain GP3087 *(rad50S),* prepared as in Figure S6A. Also shown are blots bridging *Not*I fragments where breaks were observed in the bridging blots. One DSB site was refractory to analysis. The restriction enzyme used, its length, and the fragment end probed (R or L) are indicated. The DSBs are numbered as in Figures 3 and 4.
(1.3 MB PDF)

Figure S7 Rec12-DNA Linkages across the Entire S. pombe Genome Data, filtered as in Figure S2C, are the IP:WCE ratios before induction (0 h; blue line) and 5 h after induction (red line; mean of two haploid experiments). Bars indicate the extent of the centromeric repeats. Carets indicate prominent (red) and weak (black) Rec12 peaks. Note that ∼0.5 Mb of rDNA, not shown, is at each end of Chromosome III.
(9.6 MB PDF)

Figure S8 Among Prominent Rec12 Peaks, Stronger Peaks Occur More Frequently in Large IGRs Each prominent Rec12 peak was allocated to one of four bins based on its peak integral (i.e*.,* strength). The percentage of all peaks in each bin that lie in IGRs >3 kb is indicated. Bin boundaries were chosen to allocate approximately equal numbers of peaks to each bin.
(11 KB PDF)

Figure S9 Meiotic DSB Positions and Relative Intensities are Similar in *rad50^+^* and *rad50S* Backgrounds and in *pat1^+^* and *pat1–114* Backgrounds *S. pombe pat1–114* strains were meiotically induced and analyzed for DSBs essentially as described [5]. *pat1^+^* strains were induced as described [46] without restoration of a nitrogen source at the beginning of the induction and with more vigorous agitation of cultures and use of baffled flasks. DNA from the times indicated was digested with *Not*I, separated on pulsed-field gels, and Southern hybridized using a ^32^P-labelled probe for the left end of the *Not*I-J fragment. The strains used were: (A) GP3718 (*pat1–114 rad50S*), (B) GP1979 (*pat1–114 rad50^+^*), (C) GP3124 (*pat1^+^ rad50S*), (D) GP3121 (*pat1^+^ rad50^+^*). W, well positions; 0, full-length *Not*I-J restriction fragment; 1, *mbs1;* and 2, *mbs2*. Quantitation of *mbs1* and *mbs2* at the time-point of maximal breakage is given on the right of each gel. Maximal DSB frequencies are greater in *rad50S* than *rad50^+^* inductions because of DSB accumulation in *rad50S*. Maximal DSB frequencies are reduced in *pat1^+^* compared to *pat1–114* inductions due to greater asynchrony in the *pat1^+^* inductions and a lower proportion of cells progressing through meiosis in that background.
(2.0 MB PDF)

Figure S10 Plots of Neighbor Dissimilarity (log_2_) against IP:WCE Hybridization Ratios for Each Probe of Chromosome I Using Data from the Haploid and Diploid Experiments (Strains GP6013 and GP6203) Neighbor dissimilarity is expressed by calculating the absolute value of log_2_ (probe IP:WCE ratio/neighbor IP:WCE ratio), using both neighboring probes, and taking the lesser value.
(A) 0 h, haploid.
(B) Mean 5 h, haploid.
(C) 0 h, diploid.
(D) 5 h, diploid.
Note that high IP:WCE hybridization ratios in 0-h data are spikes; they have high dissimilarity relative to either neighboring probe. For these probes, dissimilarity rises with IP:WCE ratio, indicating that the neighboring probes have low (background) ratios, unrelated to that of the spikes. These spike probes are also seen in 5-h data, but another distribution of probes with high IP:WCE ratios and low neighbor dissimilarity is seen. These probes reflect genuine Rec12-enrichment with elevated IP:WCE ratios across several contiguous probes.
(8.2 MB PDF)

Figure S11 Plots of Neighbor Dissimilarity (log_2_) against IP:WCE Hybridization Ratios for Each Probe of Chromosome I using Data from Both Haploid and Diploid Experiments (Strains GP6013 and GP6203) Data were filtered to remove isolated (single and double) linear values >2 (i.e., log_2_ >1), identified as in Figure S2C. Neighbor dissimilarity was calculated as in Figure S10.
(A) 0 h, haploid.
(B) Mean 5 h, haploid.
(C) 0 h, diploid.
(D) 5 h, diploid.
Note that, in comparison to Figure S10, spikes have largely been removed but genuine Rec12-enrichment peaks remain.
(7.8 MB PDF)

Table S1 *rec12-FLAG* and *rec12-HA* Are Recombination Proficient (26 KB DOC)

Table S2 Correlation of DSB Hotspots and GO Classes (31 KB DOC)

Table S3 S. pombe Strains (41 KB DOC)

Table S4 Primers for Plasmid and Allele Construction and PCR Analysis (34 KB DOC)

Text S1 Construction of pJF32 *(rec12-201::6His-2FLAG)* and pJF41 *(rec12-202::6His-2HA)*
(23 KB DOC)

## Abbreviations

DSB: double-strand break
GC: guanine-cytosine
GO: gene ontology
IGR: intergenic region
IP: immunoprecipitate
WCE: whole cell extract

## Note Added in Proof

Reference 24 is cited out of order.
