# Supplementary material for: A Discrete Class of Intergenic DNA Dictates Meiotic DNA Break Hotspots in Fission Yeast
Source: PLoS Genet. 2007 Aug 24;3(8):e141. doi: 10.1371/journal.pgen.0030141 (PMC1950956; doi:10.1371/journal.pgen.0030141)
Supplement: Supplementary file 1 [file pgen.0030141.sg001.pdf]

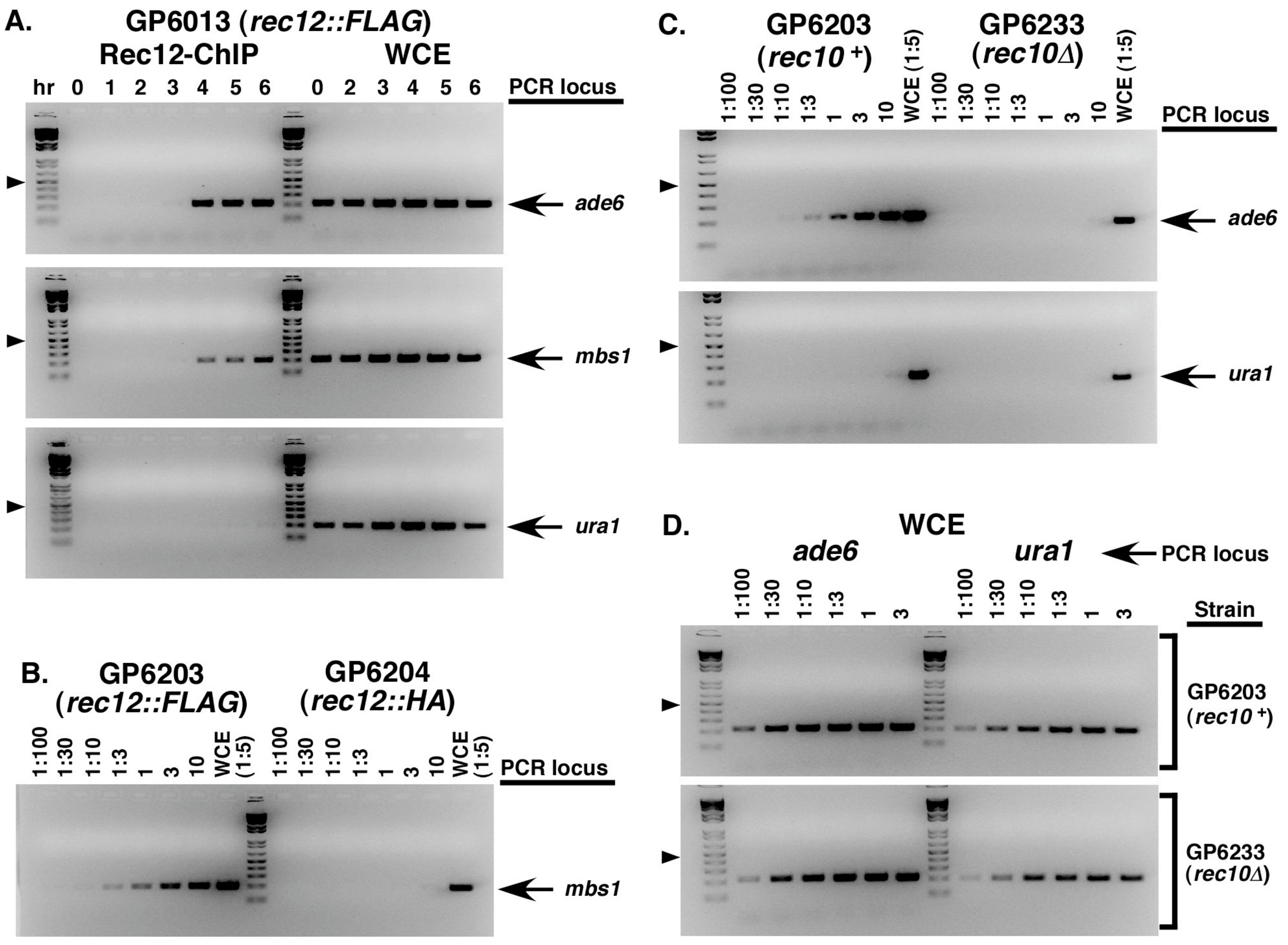
A.
GP6013 (rec12::FLAG)
Rec12-ChIP
WCE
hr 0 1 2 3 4 5 6
0 2 3 4 5 6
PCR locus
ade6
mbs1
ura1
B.
GP6203
(rec12::FLAG)
GP6204
(rec12::HA)
1:100 1:30 1:10 1:3 1 3 10 WCE (1:5)
1:100 1:30 1:10 1:3 1 3 10 WCE (1:5)
PCR locus
mbs1
C.
GP6203
(rec10 +)
GP6233
(rec10Δ)
1:100 1:30 1:10 1:3 1 3 10 WCE (1:5)
1:100 1:30 1:10 1:3 1 3 10 WCE (1:5)
PCR locus
ade6
ura1
D.
WCE
ade6
ura1
PCR locus
1:100 1:30 1:10 1:3 1 3
1:100 1:30 1:10 1:3 1 3
Strain
GP6203
(rec10 +)
GP6233
(rec10Δ)
