# Supplementary material for: A Discrete Class of Intergenic DNA Dictates Meiotic DNA Break Hotspots in Fission Yeast
Source: PLoS Genet. 2007 Aug 24;3(8):e141. doi: 10.1371/journal.pgen.0030141 (PMC1950956; doi:10.1371/journal.pgen.0030141)
Supplement: Supplementary file 2 [file pgen.0030141.sg002.pdf]

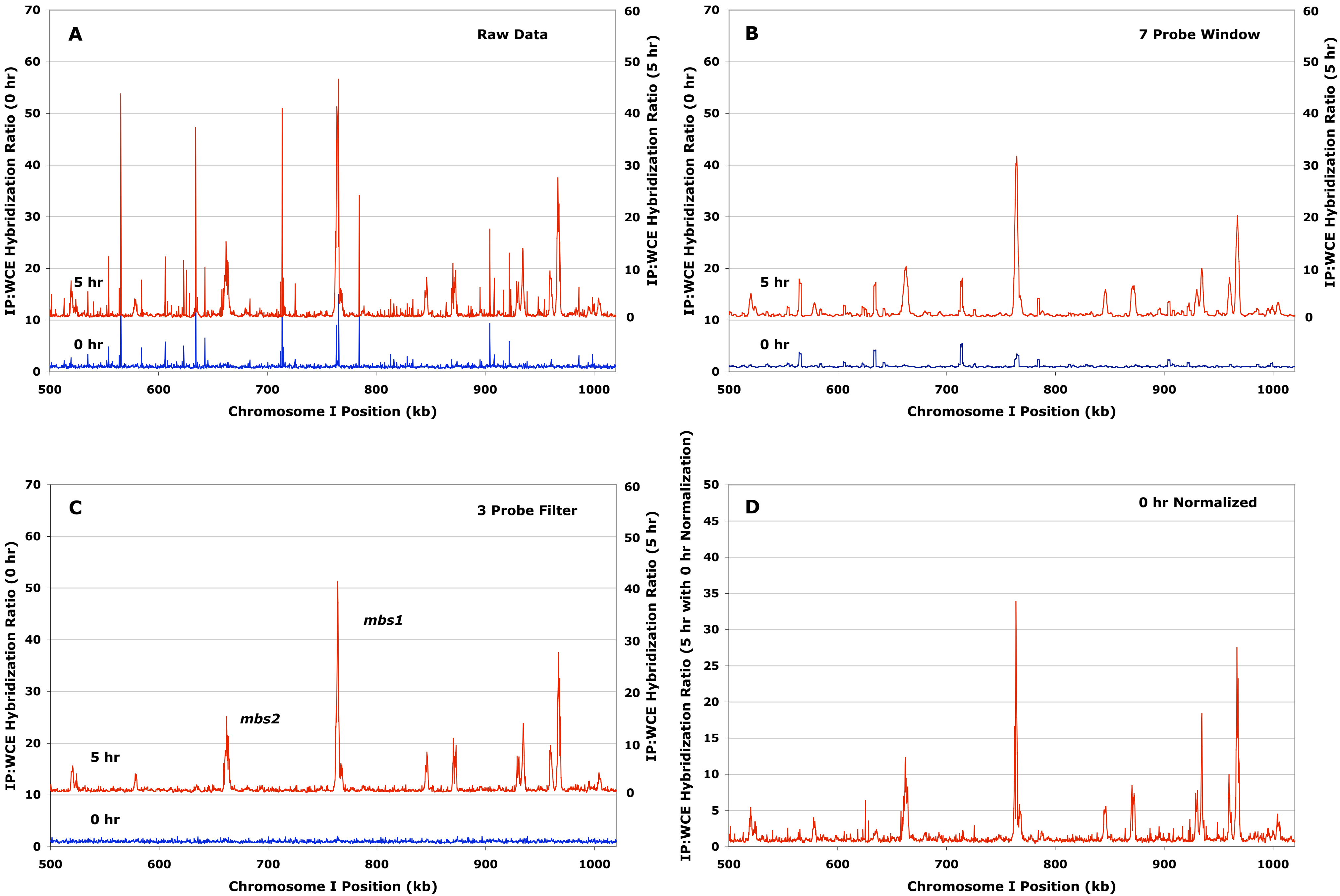

A
Raw Data
5 hr
0 hr
B
7 Probe Window
C
3 Probe Filter
mbs1
mbs2
D
0 hr Normalized
IP:WCE Hybridization Ratio (0 hr)
IP:WCE Hybridization Ratio (5 hr)
IP:WCE Hybridization Ratio (5 hr with 0 hr Normalization)
Chromosome I Position (kb)
