# Supplementary material for: A Discrete Class of Intergenic DNA Dictates Meiotic DNA Break Hotspots in Fission Yeast
Source: PLoS Genet. 2007 Aug 24;3(8):e141. doi: 10.1371/journal.pgen.0030141 (PMC1950956; doi:10.1371/journal.pgen.0030141)
Supplement: Supplementary file 3 [file pgen.0030141.sg003.pdf]

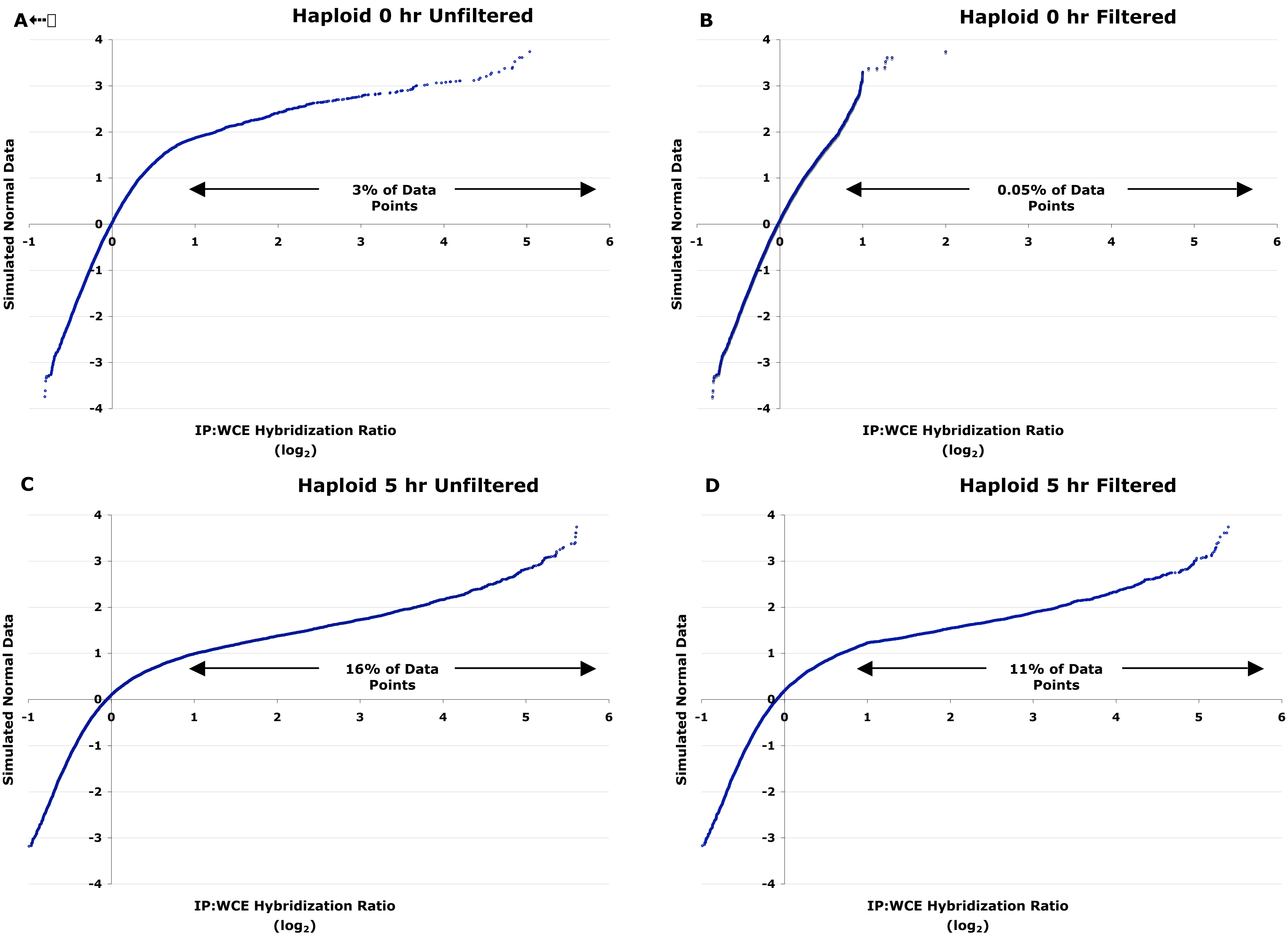
A
Haploid 0 hr Unfiltered
Simulated Normal Data
3% of Data Points
IP:WCE Hybridization Ratio (log$_2$)
B
Haploid 0 hr Filtered
Simulated Normal Data
0.05% of Data Points
IP:WCE Hybridization Ratio (log$_2$)
C
Haploid 5 hr Unfiltered
Simulated Normal Data
16% of Data Points
IP:WCE Hybridization Ratio (log$_2$)
D
Haploid 5 hr Filtered
Simulated Normal Data
11% of Data Points
IP:WCE Hybridization Ratio (log$_2$)
