# Supplementary material for: A Discrete Class of Intergenic DNA Dictates Meiotic DNA Break Hotspots in Fission Yeast
Source: PLoS Genet. 2007 Aug 24;3(8):e141. doi: 10.1371/journal.pgen.0030141 (PMC1950956; doi:10.1371/journal.pgen.0030141)
Supplement: Supplementary file 4 [file pgen.0030141.sg004.pdf]

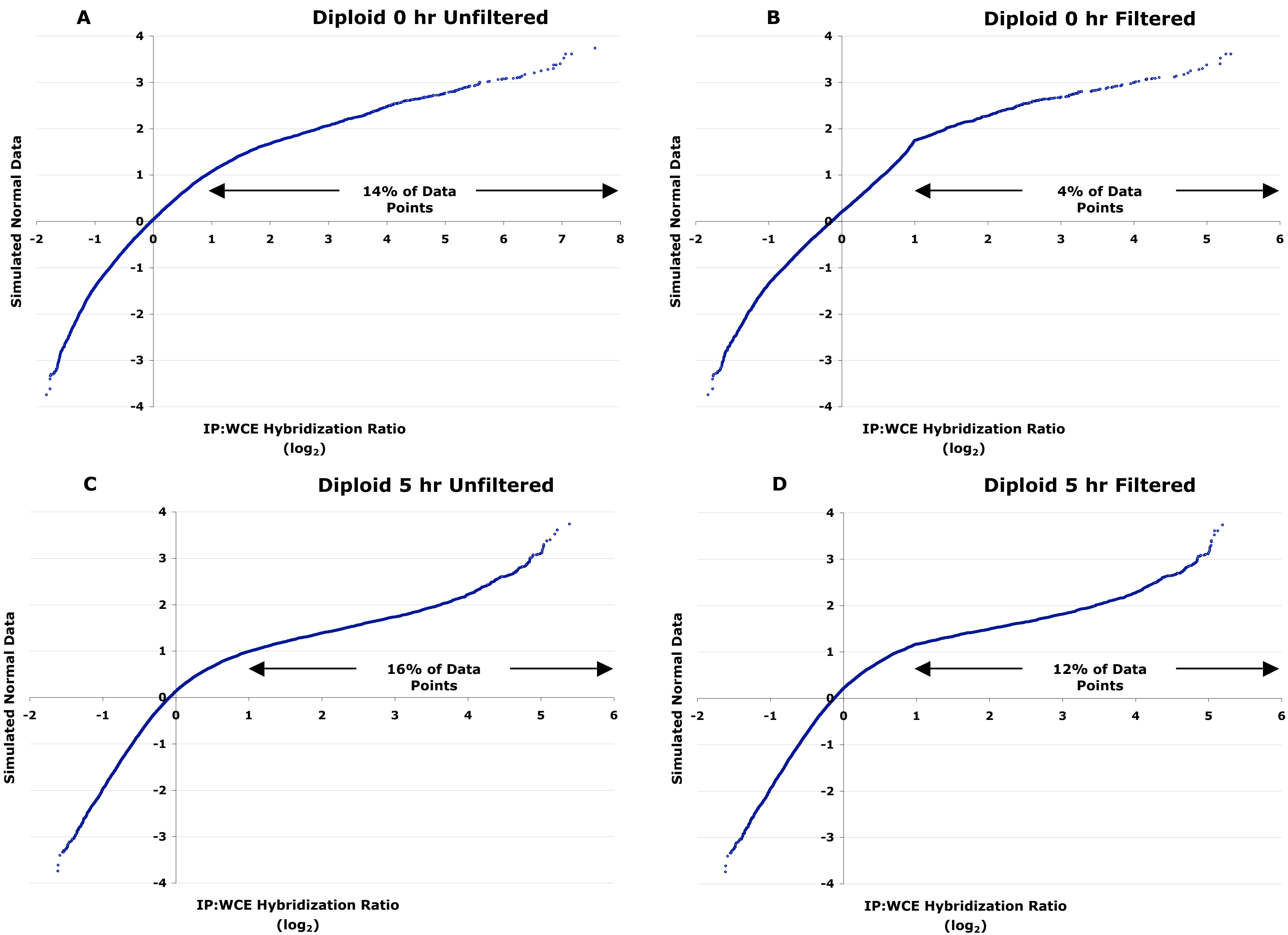

A
Diploid 0 hr Unfiltered
14% of Data Points
B
Diploid 0 hr Filtered
4% of Data Points
C
Diploid 5 hr Unfiltered
16% of Data Points
D
Diploid 5 hr Filtered
12% of Data Points
Simulated Normal Data
IP:WCE Hybridization Ratio ($\log_2$)
