# Supplementary material for: A Discrete Class of Intergenic DNA Dictates Meiotic DNA Break Hotspots in Fission Yeast
Source: PLoS Genet. 2007 Aug 24;3(8):e141. doi: 10.1371/journal.pgen.0030141 (PMC1950956; doi:10.1371/journal.pgen.0030141)
Supplement: Supplementary file 5 [file pgen.0030141.sg005.pdf]

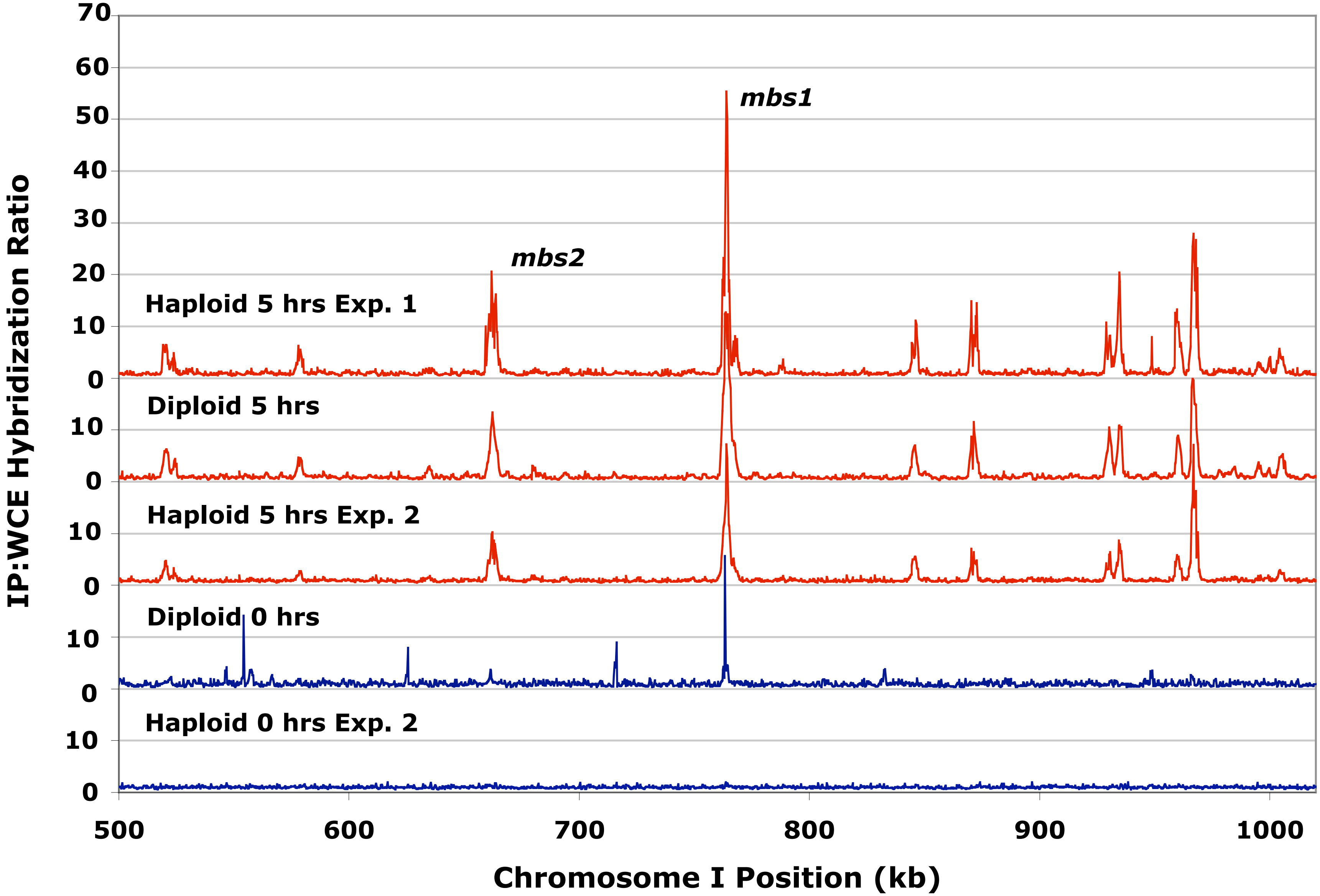

mbs1
mbs2
Haploid 5 hrs Exp. 1
Diploid 5 hrs
Haploid 5 hrs Exp. 2
Diploid 0 hrs
Haploid 0 hrs Exp. 2
IP:WCE Hybridization Ratio
70
60
50
40
30
20
10
0
500
600
700
800
900
1000
Chromosome I Position (kb)
