# Supplementary material for: A Discrete Class of Intergenic DNA Dictates Meiotic DNA Break Hotspots in Fission Yeast
Source: PLoS Genet. 2007 Aug 24;3(8):e141. doi: 10.1371/journal.pgen.0030141 (PMC1950956; doi:10.1371/journal.pgen.0030141)
Supplement: Supplementary file 6 [file pgen.0030141.sg006.pdf]

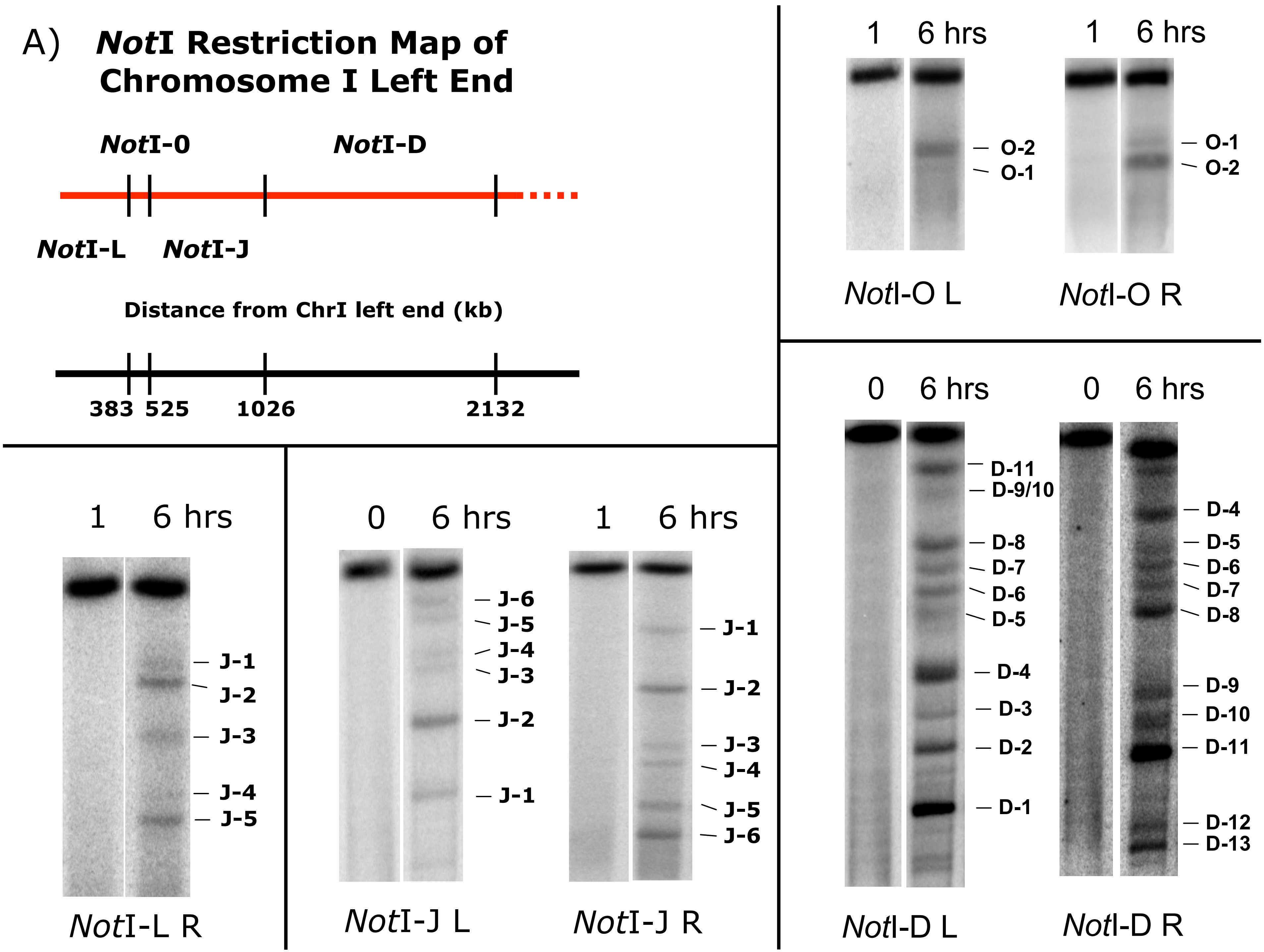

A) NotI Restriction Map of Chromosome I Left End
NotI-0
NotI-D
NotI-L
NotI-J
Distance from ChrI left end (kb)
383
525
1026
2132
1
6 hrs
O-2
O-1
NotI-O L
1
6 hrs
O-1
O-2
NotI-O R
0
6 hrs
D-11
D-9/10
D-8
D-7
D-6
D-5
D-4
D-3
D-2
D-1
NotI-D L
0
6 hrs
D-4
D-5
D-6
D-7
D-8
D-9
D-10
D-11
D-12
D-13
NotI-D R
1
6 hrs
J-1
J-2
J-3
J-4
J-5
NotI-L R
0
6 hrs
J-6
J-5
J-4
J-3
J-2
J-1
NotI-J L
1
6 hrs
J-1
J-2
J-3
J-4
J-5
J-6
NotI-J R

B)

DSB Hotspot from *Not*I Blots	Distance from Chr. I Left End (kb)
L1	<200
L2	<200
L3	<200
L4	215
L5	240
O1	440
O2	460
J1	655
J2	760
J3	850
J4	870
J5	930
J6	960
D1	1140
D2	1230
D3	1285
D4	1365
D5	1480
D6	1540
D7	1600
D8	1675
D9	1865
D10	1915
D11	1970
D12	>2000
D13	>2000

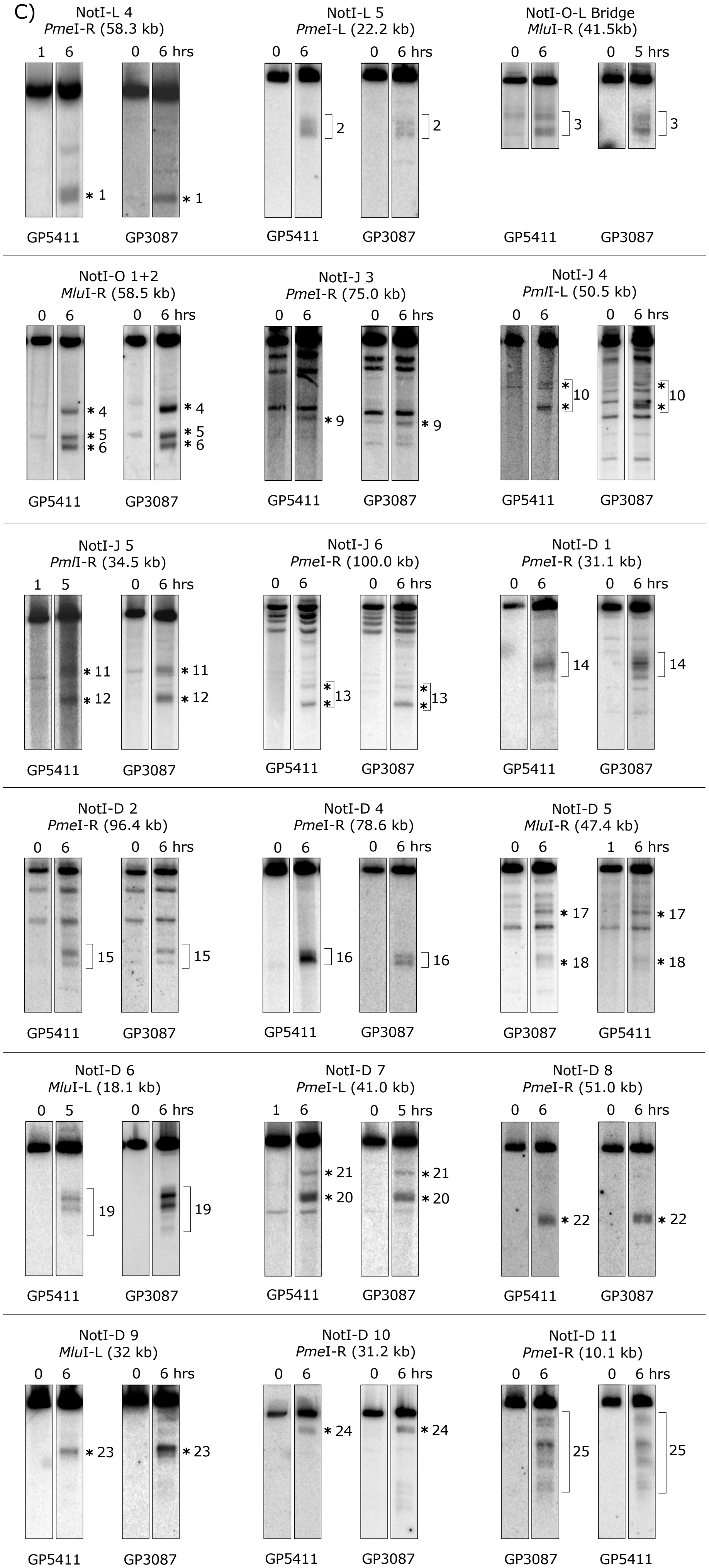
C)
NotI-L 4
PmeI-R (58.3 kb)
1 6 0 6 hrs
* 1
* 1
GP5411 GP3087
NotI-L 5
PmeI-L (22.2 kb)
0 6 0 6 hrs
2
2
GP5411 GP3087
NotI-O-L Bridge
MluI-R (41.5kb)
0 6 0 5 hrs
3
3
GP5411 GP3087
NotI-O 1+2
MluI-R (58.5 kb)
0 6 0 6 hrs
* 4
* 5
* 6
GP5411 GP3087
NotI-J 3
PmeI-R (75.0 kb)
0 6 0 6 hrs
* 9
* 9
GP5411 GP3087
NotI-J 4
PmlI-L (50.5 kb)
0 6 0 6 hrs
10
10
GP5411 GP3087
NotI-J 5
PmlI-R (34.5 kb)
1 5 0 6 hrs
* 11
* 12
GP5411 GP3087
NotI-J 6
PmeI-R (100.0 kb)
0 6 0 6 hrs
13
13
GP5411 GP3087
NotI-D 1
PmeI-R (31.1 kb)
0 6 0 6 hrs
14
14
GP5411 GP3087
NotI-D 2
PmeI-R (96.4 kb)
0 6 0 6 hrs
15
15
GP5411 GP3087
NotI-D 4
PmeI-R (78.6 kb)
0 6 0 6 hrs
16
16
GP5411 GP3087
NotI-D 5
MluI-R (47.4 kb)
0 6 1 6 hrs
* 17
* 18
GP3087 GP5411
NotI-D 6
MluI-L (18.1 kb)
0 5 0 6 hrs
19
19
GP5411 GP3087
NotI-D 7
PmeI-L (41.0 kb)
1 6 0 5 hrs
* 21
* 20
GP5411 GP3087
NotI-D 8
PmeI-R (51.0 kb)
0 6 0 6 hrs
* 22
* 22
GP5411 GP3087
NotI-D 9
MluI-L (32 kb)
0 6 0 6 hrs
* 23
* 23
GP5411 GP3087
NotI-D 10
PmeI-R (31.2 kb)
0 6 0 6 hrs
* 24
* 24
GP5411 GP3087
NotI-D 11
PmeI-R (10.1 kb)
0 6 0 6 hrs
25
25
GP3087 GP5411
