# Supplementary material for: A Discrete Class of Intergenic DNA Dictates Meiotic DNA Break Hotspots in Fission Yeast
Source: PLoS Genet. 2007 Aug 24;3(8):e141. doi: 10.1371/journal.pgen.0030141 (PMC1950956; doi:10.1371/journal.pgen.0030141)
Supplement: Supplementary file 7 [file pgen.0030141.sg007.pdf]

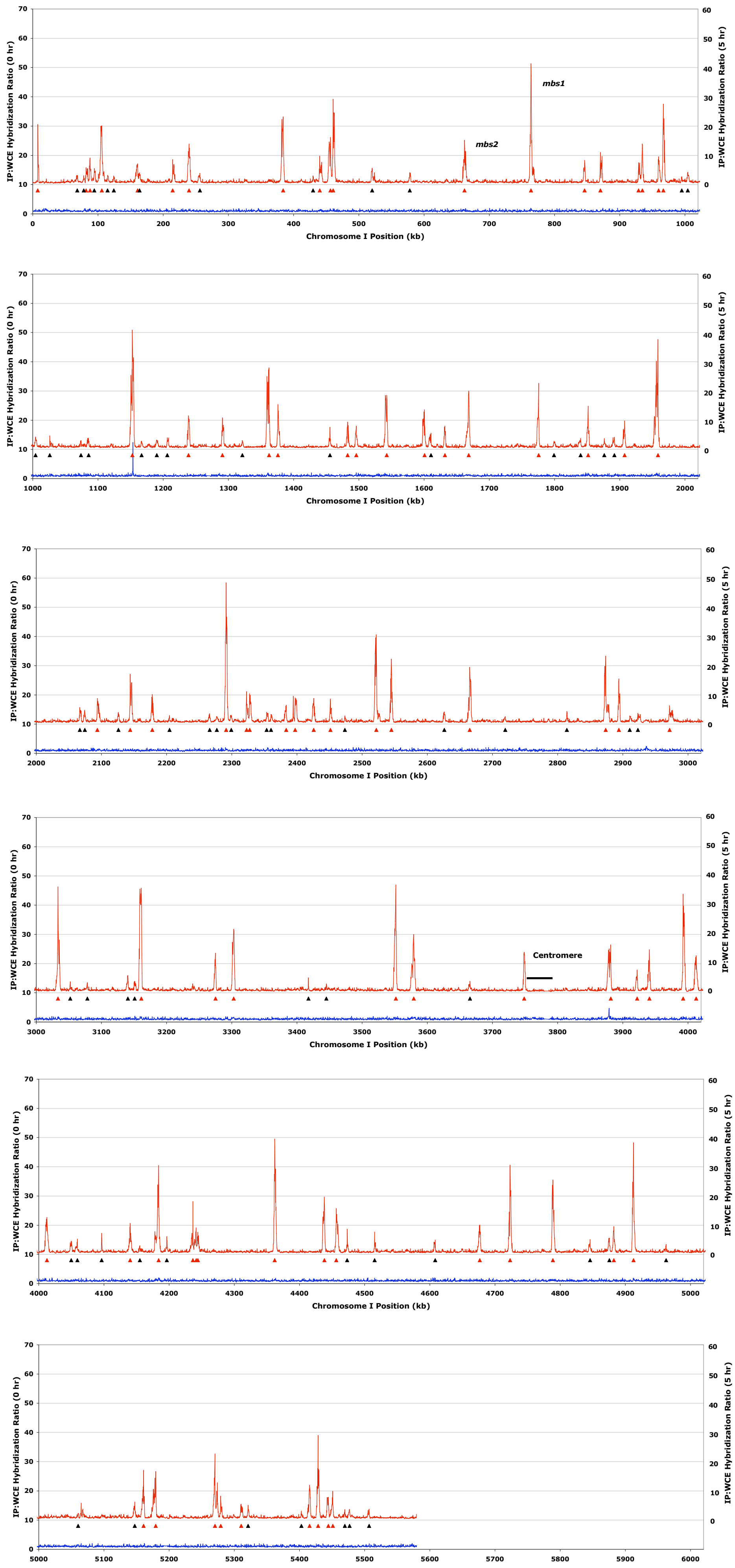

IP:WCE Hybridization Ratio (0 hr)
IP:WCE Hybridization Ratio (5 hr)
mbs1
mbs2
Chromosome I Position (kb)
IP:WCE Hybridization Ratio (0 hr)
IP:WCE Hybridization Ratio (5 hr)
Chromosome I Position (kb)
IP:WCE Hybridization Ratio (0 hr)
IP:WCE Hybridization Ratio (5 hr)
Chromosome I Position (kb)
IP:WCE Hybridization Ratio (0 hr)
IP:WCE Hybridization Ratio (5 hr)
Centromere
Chromosome I Position (kb)
IP:WCE Hybridization Ratio (0 hr)
IP:WCE Hybridization Ratio (5 hr)
Chromosome I Position (kb)
IP:WCE Hybridization Ratio (0 hr)
IP:WCE Hybridization Ratio (5 hr)
Chromosome I Position (kb)

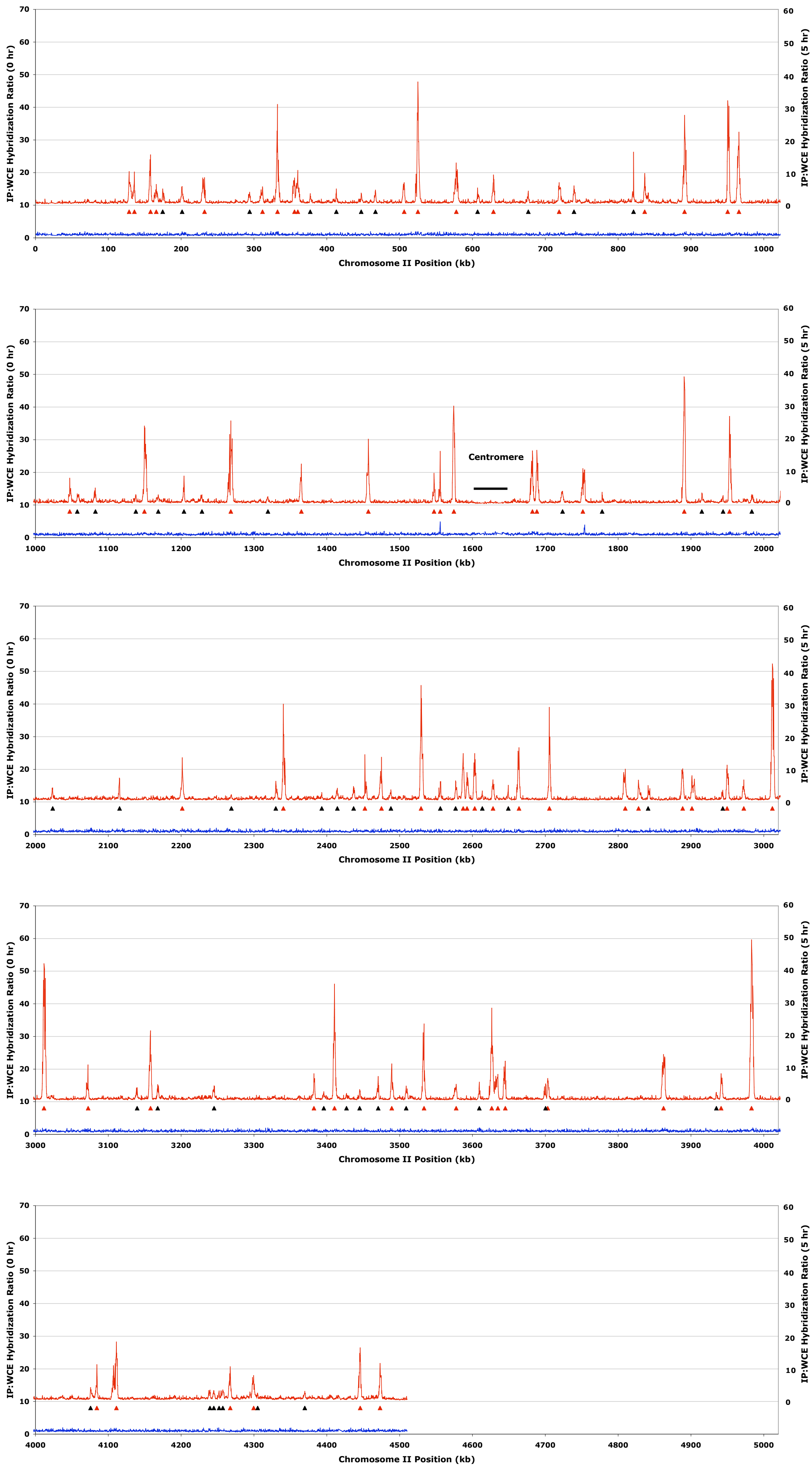
IP:WCE Hybridization Ratio (0 hr)
IP:WCE Hybridization Ratio (5 hr)
Chromosome II Position (kb)
Centromere

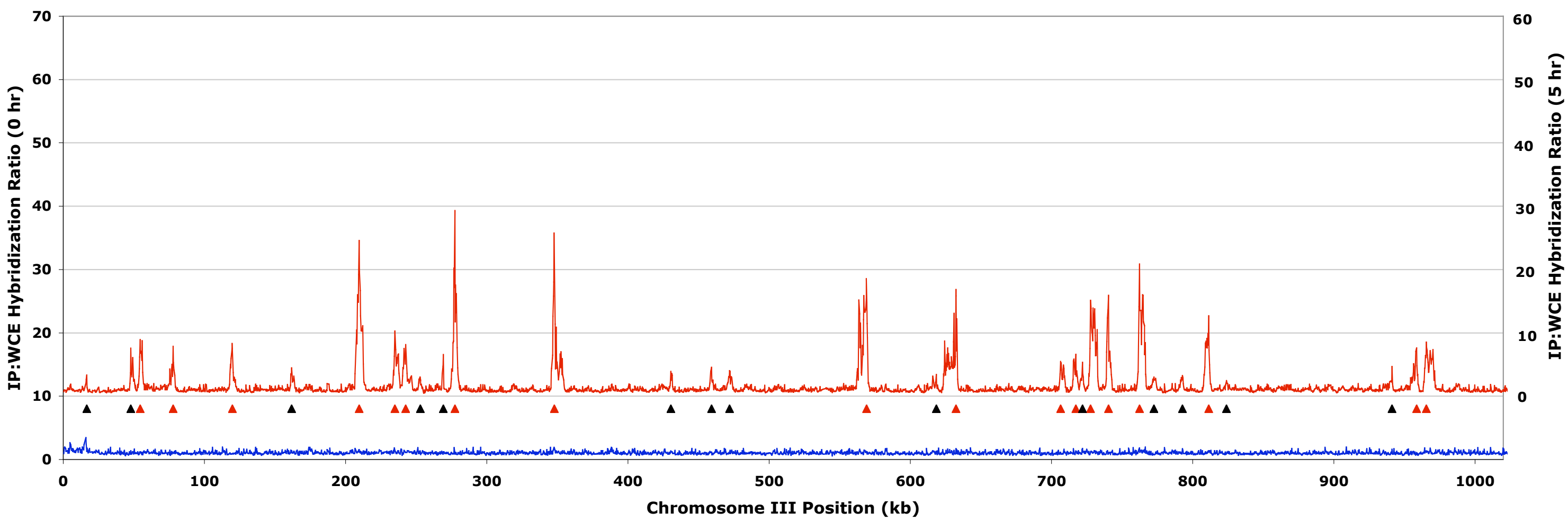
IP:WCE Hybridization Ratio (0 hr)
IP:WCE Hybridization Ratio (5 hr)
Chromosome III Position (kb)

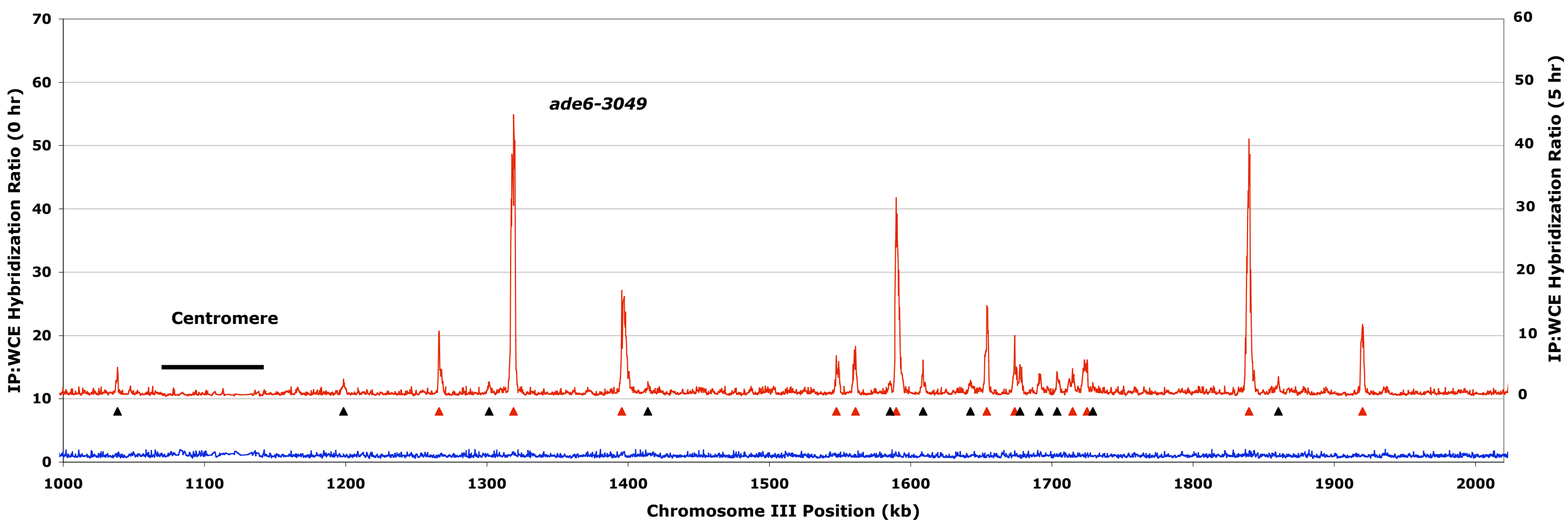
ade6-3049
Centromere
IP:WCE Hybridization Ratio (0 hr)
IP:WCE Hybridization Ratio (5 hr)
Chromosome III Position (kb)

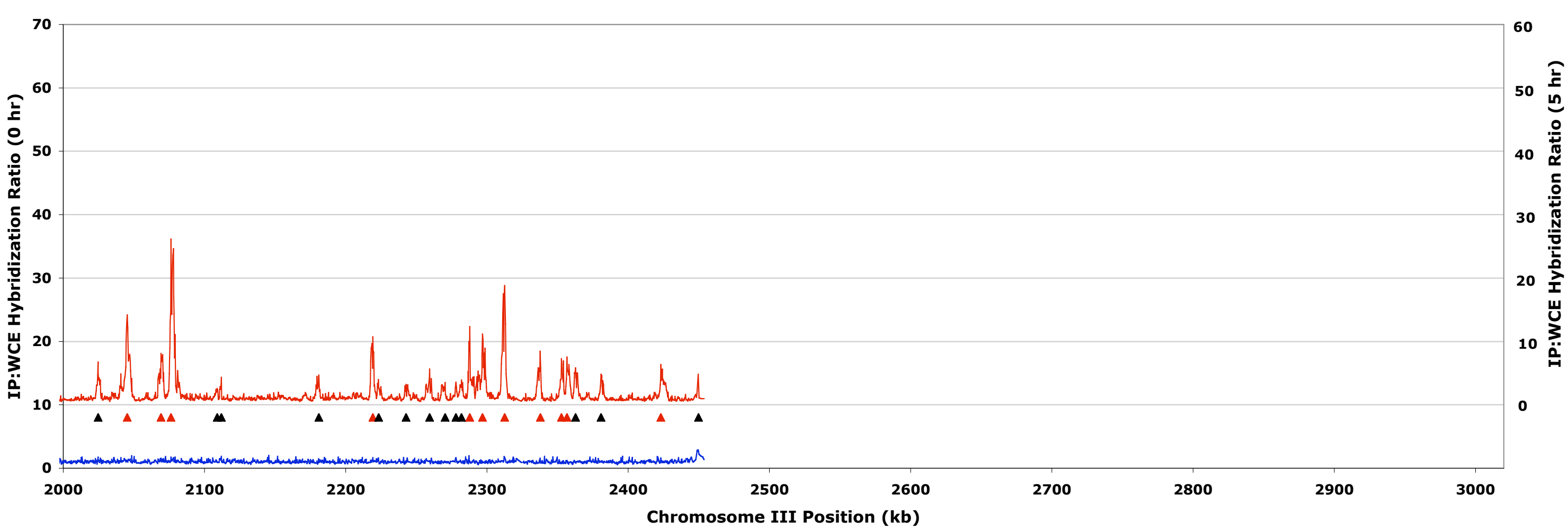
IP:WCE Hybridization Ratio (0 hr)
IP:WCE Hybridization Ratio (5 hr)
Chromosome III Position (kb)
