# Supplementary material for: A Discrete Class of Intergenic DNA Dictates Meiotic DNA Break Hotspots in Fission Yeast
Source: PLoS Genet. 2007 Aug 24;3(8):e141. doi: 10.1371/journal.pgen.0030141 (PMC1950956; doi:10.1371/journal.pgen.0030141)
Supplement: Supplementary file 8 [file pgen.0030141.sg008.pdf]

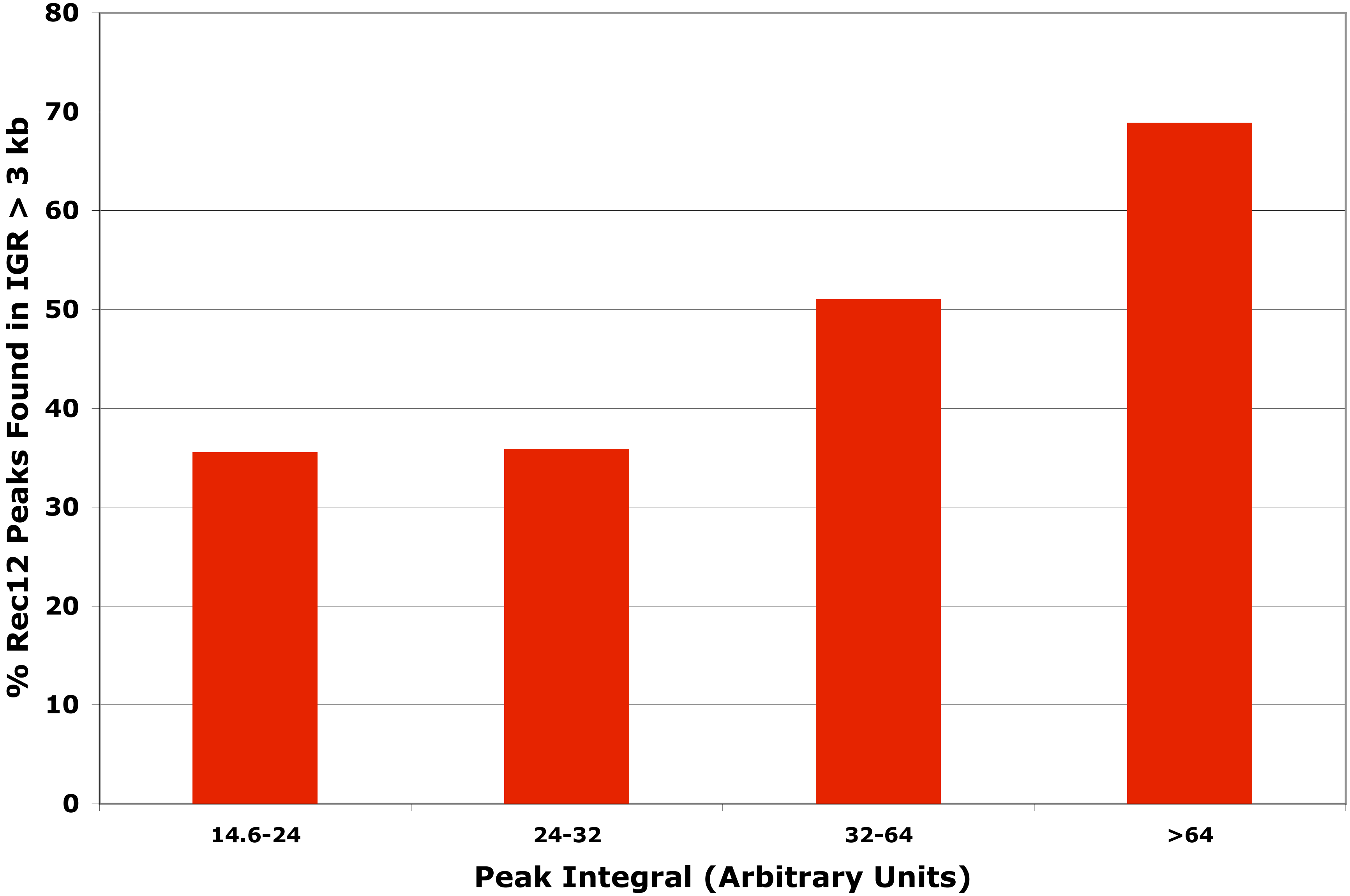
80
70
60
50
40
30
20
10
0
% Rec12 Peaks Found in IGR > 3 kb
14.6-24
24-32
32-64
>64
Peak Integral (Arbitrary Units)
