# Supplementary material for: A Discrete Class of Intergenic DNA Dictates Meiotic DNA Break Hotspots in Fission Yeast
Source: PLoS Genet. 2007 Aug 24;3(8):e141. doi: 10.1371/journal.pgen.0030141 (PMC1950956; doi:10.1371/journal.pgen.0030141)
Supplement: Supplementary file 9 [file pgen.0030141.sg009.pdf]

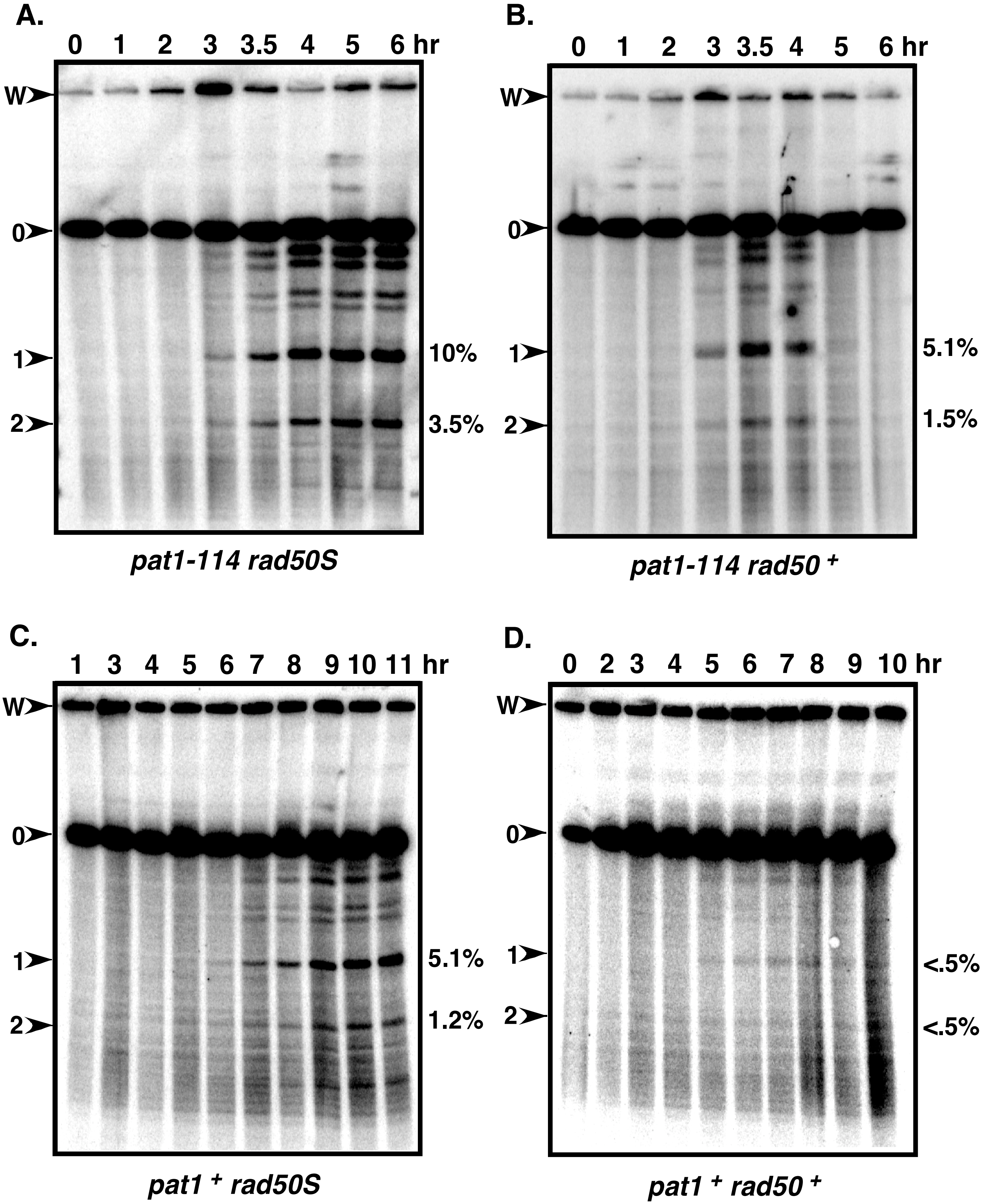

A.
0 1 2 3 3.5 4 5 6 hr
W
0
1
2
10%
3.5%
pat1-114 rad50S
B.
0 1 2 3 3.5 4 5 6 hr
W
0
1
2
5.1%
1.5%
pat1-114 rad50 +
C.
1 3 4 5 6 7 8 9 10 11 hr
W
0
1
2
5.1%
1.2%
pat1 + rad50S
D.
0 2 3 4 5 6 7 8 9 10 hr
W
0
1
2
<.5%
<.5%
pat1 + rad50 +
