# Supplementary material for: A Discrete Class of Intergenic DNA Dictates Meiotic DNA Break Hotspots in Fission Yeast
Source: PLoS Genet. 2007 Aug 24;3(8):e141. doi: 10.1371/journal.pgen.0030141 (PMC1950956; doi:10.1371/journal.pgen.0030141)
Supplement: Supplementary file 10 [file pgen.0030141.sg010.pdf]

A

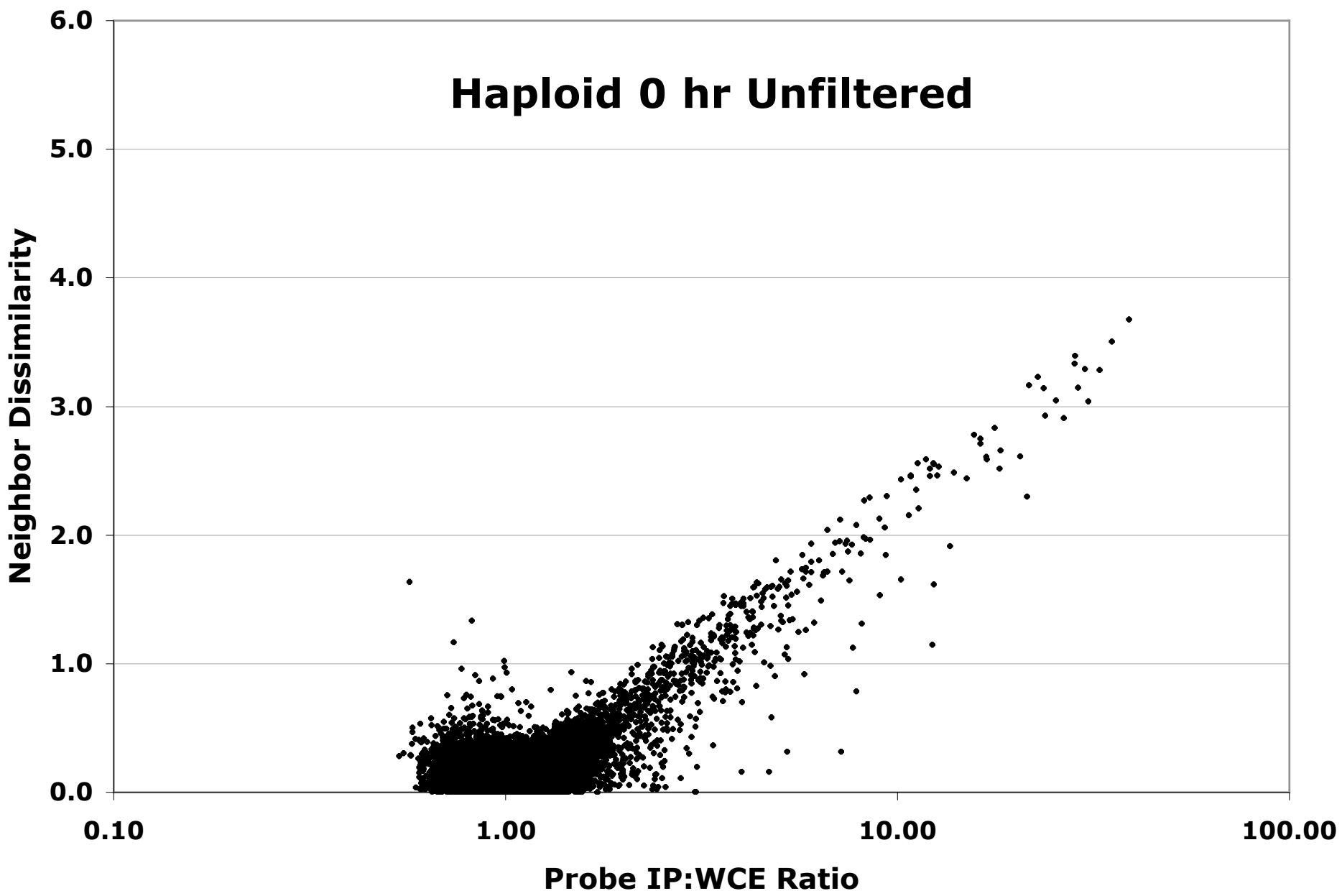
Haploid 0 hr Unfiltered
Neighbor Dissimilarity
Probe IP:WCE Ratio
0.0
1.0
2.0
3.0
4.0
5.0
6.0
0.10
1.00
10.00
100.00

B

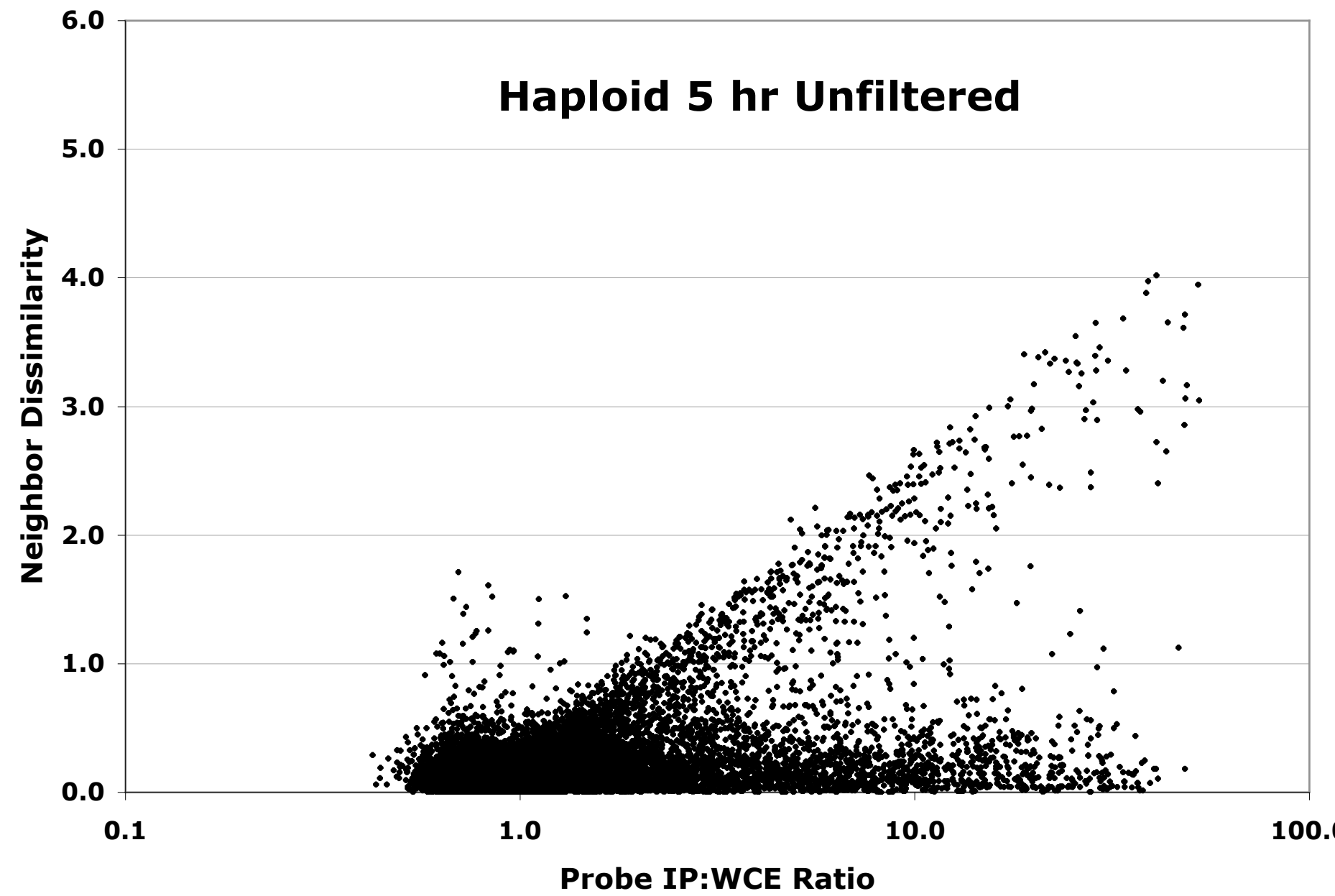
Haploid 5 hr Unfiltered
Neighbor Dissimilarity
Probe IP:WCE Ratio
0.0
1.0
2.0
3.0
4.0
5.0
6.0
0.1
1.0
10.0
100.0

C

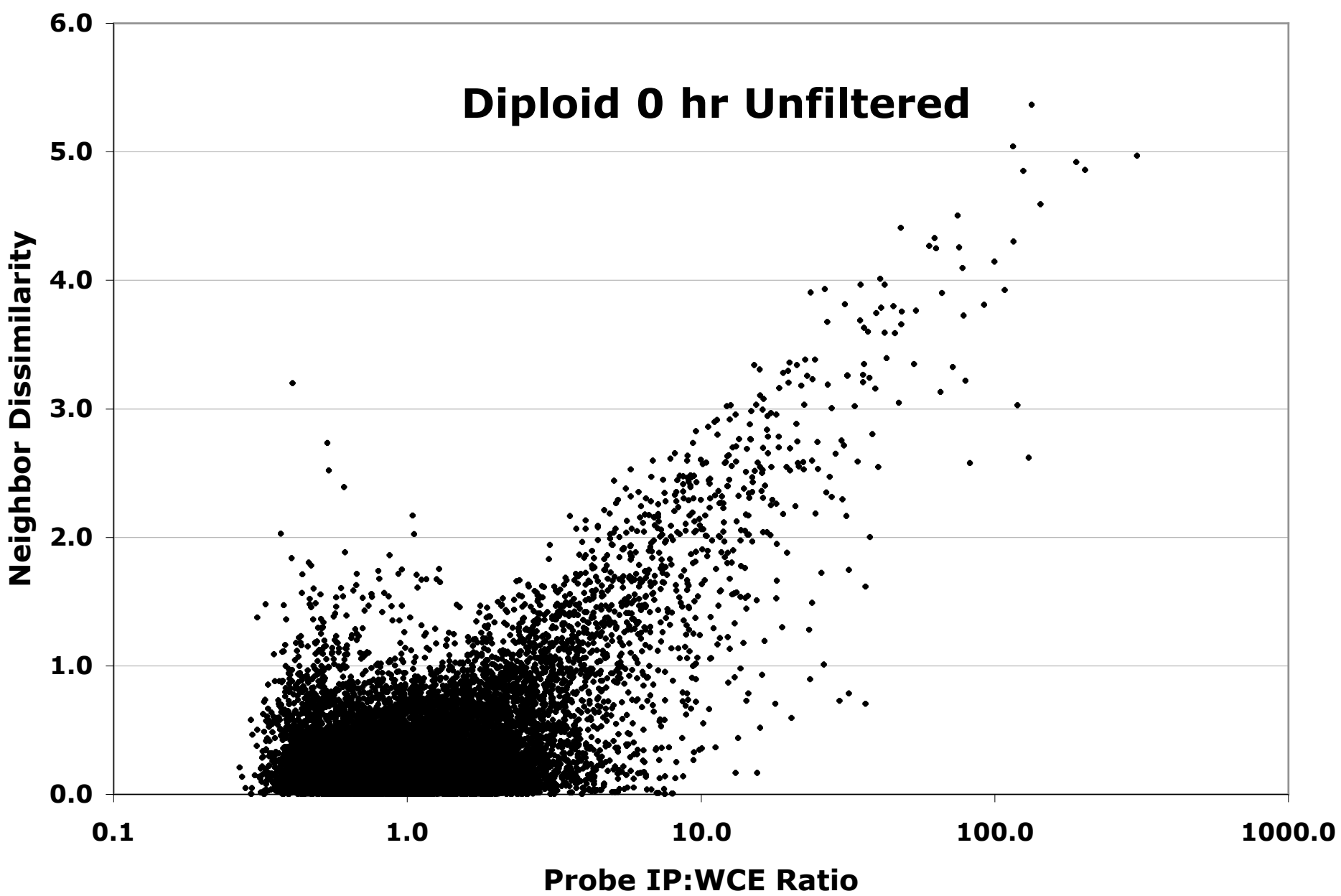
Diploid 0 hr Unfiltered
Neighbor Dissimilarity
Probe IP:WCE Ratio
0.0
1.0
2.0
3.0
4.0
5.0
6.0
0.1
1.0
10.0
100.0
1000.0

D

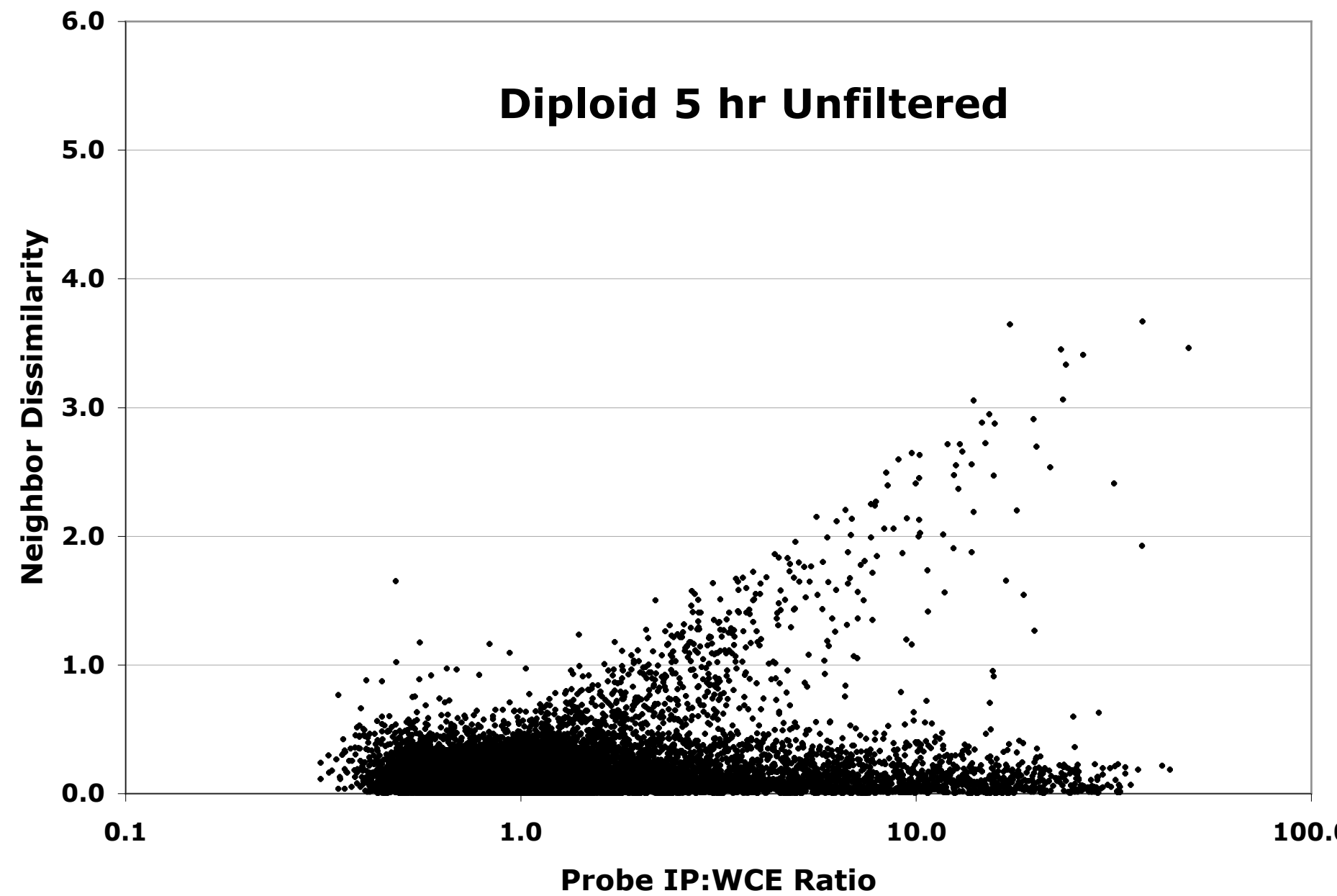
Diploid 5 hr Unfiltered
Neighbor Dissimilarity
Probe IP:WCE Ratio
0.0
1.0
2.0
3.0
4.0
5.0
6.0
0.1
1.0
10.0
100.0
