# Supplementary material for: A Discrete Class of Intergenic DNA Dictates Meiotic DNA Break Hotspots in Fission Yeast
Source: PLoS Genet. 2007 Aug 24;3(8):e141. doi: 10.1371/journal.pgen.0030141 (PMC1950956; doi:10.1371/journal.pgen.0030141)
Supplement: Supplementary file 11 [file pgen.0030141.sg011.pdf]

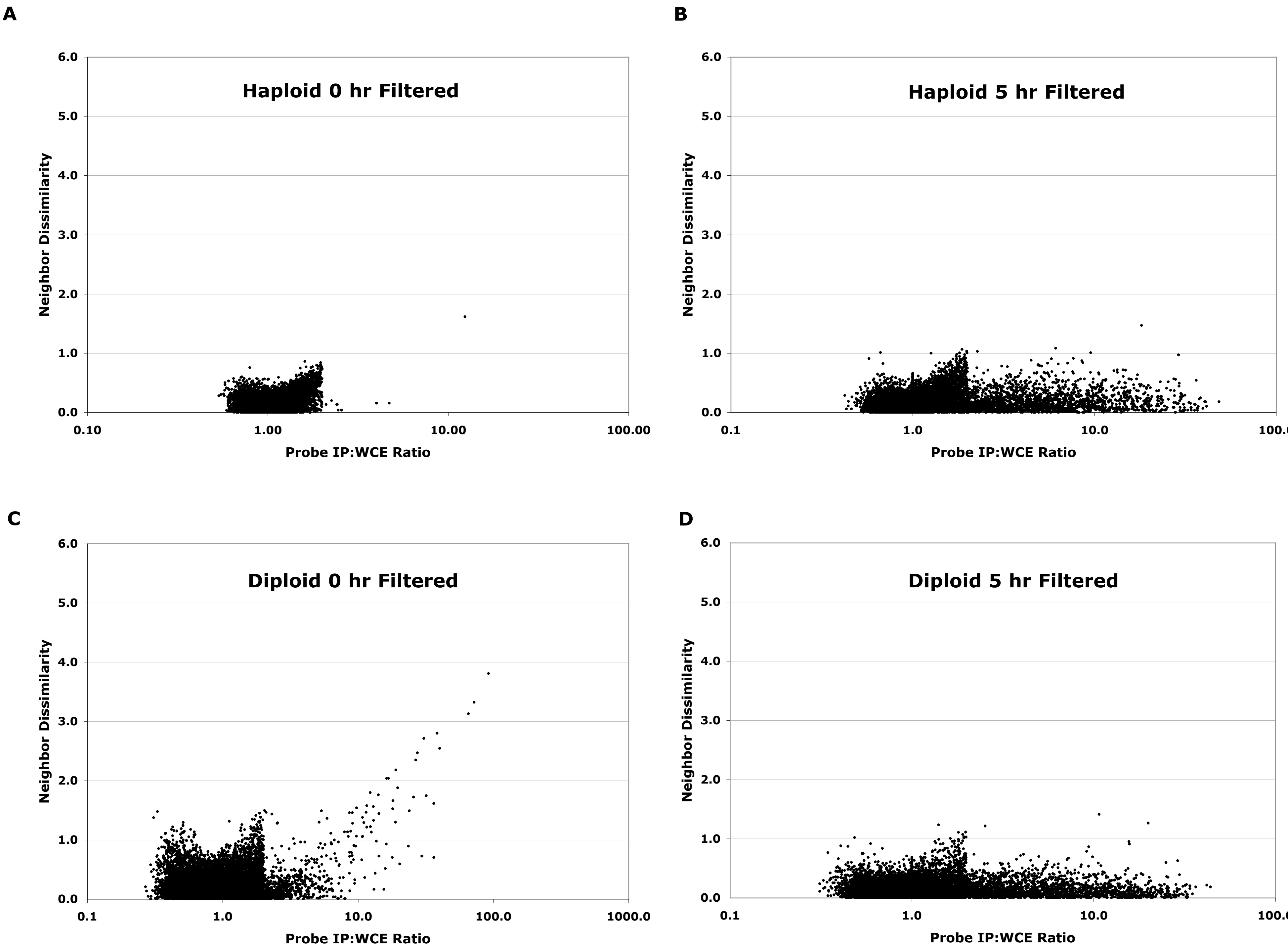
A
Haploid 0 hr Filtered
Neighbor Dissimilarity
6.0
5.0
4.0
3.0
2.0
1.0
0.0
0.10
1.00
10.00
100.00
Probe IP:WCE Ratio
B
Haploid 5 hr Filtered
Neighbor Dissimilarity
6.0
5.0
4.0
3.0
2.0
1.0
0.0
0.1
1.0
10.0
100.0
Probe IP:WCE Ratio
C
Diploid 0 hr Filtered
Neighbor Dissimilarity
6.0
5.0
4.0
3.0
2.0
1.0
0.0
0.1
1.0
10.0
100.0
1000.0
Probe IP:WCE Ratio
D
Diploid 5 hr Filtered
Neighbor Dissimilarity
6.0
5.0
4.0
3.0
2.0
1.0
0.0
0.1
1.0
10.0
100.0
Probe IP:WCE Ratio
