# Supplementary material for: A Discrete Class of Intergenic DNA Dictates Meiotic DNA Break Hotspots in Fission Yeast
Source: PLoS Genet. 2007 Aug 24;3(8):e141. doi: 10.1371/journal.pgen.0030141 (PMC1950956; doi:10.1371/journal.pgen.0030141)
Supplement: Supplementary file 12 [file pgen.0030141.sd003.doc]

**Cromie *et al.*, Supporting Information**

**Construction of pJF32 (*rec12-201::6His-2FLAG*) and pJF41 (*rec12::202-6His-2HA*)**

The *rec12*-coding part of plasmid pYL78 *rec*^1^2+ (Lin and Smith, 1994) was replaced with *rec12* cDNA to generate pYL182 as follows (Y. Lin and G. Smith, unpublished data). The cDNA was generated by reverse transcription of RNA isolated 0 – 6 hr after meiotic induction of strain GP34 (*h^+^ ade6-M26 pat1-114*) using primers oYL1 and oYL2 (Table S4). The 860-bp product was purified, and a portion reamplified with the same primers; the product was digested with *Eco*RI. This 834-bp fragment was ligated to the large (8.2 kb) *Eco*RI fragment of pYL121, an ExoIII-generated derivative of pYL78 lacking ~ 0.3 kb at the 3’ end of the *rec12* fragment (Lin and Smith, 1994). pYL182 contains ~2.0 kb 5’ and ~ 0.2 kb 3’ of the *rec12* intronless ORF.

Plasmid pYL182 was used as template in a PCR with primers oJF08 and oJF09. The 1.5 kb product was digested with *Bam*HI and inserted into the *Bam*HI site of pBluescript KS(+) (Stratagene) to give pJF17. DNA encoding six histidine residues was inserted immediately after the last codon of *rec12* in pJF17 using the Morph kit (5’ → 3’, Inc.) and oJF12 as primer, to give pJF19. The 0.5 kb *NotI* – *Nco*I fragment of pJF19, made blunt-ended at the *Nco*I site with the Klenow fragment of DNA polymerase I and containing the 3’ part of *rec12*, was inserted between the *Not*I and *Eco*RV sites of pBluescript KS(+) to give pJF30. Annealed oligonucleotides oJF21 and oJF22 were inserted into the *Mlu*I site of pJF30 to give pJF31. The 0.5 kb *Nsi*I – *Nco*I fragment of pJF31 was swapped with the corresponding fragment of pJF17 to give pJF32.

Annealed oligonucleotides oJF27 and oJF28 were inserted into the *Mlu*I site of pJF30 to give pJF35. The 0.5 kb *Nsi*I – *Not*I fragment of pJF35 was swapped with the corresponding fragment of pJF17 to give pJF41.
