# Supplementary material for: A Discrete Class of Intergenic DNA Dictates Meiotic DNA Break Hotspots in Fission Yeast
Source: PLoS Genet. 2007 Aug 24;3(8):e141. doi: 10.1371/journal.pgen.0030141 (PMC1950956; doi:10.1371/journal.pgen.0030141)
Supplement: Supplementary file 13 [file pgen.0030141.st001.doc]

**Table S1**

***rec12-FLAG* and *rec12-HA*** are recombination-proficient

| Strains crossed | *rec12* allele in parents | Ade^+^  spores/l0^3^ viable spores |
| --- | --- | --- |
| GP4999 x GP5387 | +/Δ | 18.2 ± 1.7 |
| GP5960 x GP5387 | *rec12-201::6His-2FLAG*/Δ | 17.9 ± 2.6 |
| GP5961 x GP5387 | *rec12-202::6His-2HA*/Δ | 16.3 ± 0.6 |

The indicated strains, bearing the *ade6-3049* and *ade6-M26* alleles, were crossed, and the frequency of Ade^+^ recombinants among total viable spores was determined as described (Steiner and Smith, 2005). Each datum is the mean ± SEM of one cross on one day and four crosses on another day.

References:

Steiner, W. W., and Smith, G. R. (2005). Optimizing the nucleotide sequence of a meiotic recombination hotspot in *Schizosaccharomyces pombe*. *Genetics* **169**, 1973-1983.
