# Supplementary material for: A Discrete Class of Intergenic DNA Dictates Meiotic DNA Break Hotspots in Fission Yeast
Source: PLoS Genet. 2007 Aug 24;3(8):e141. doi: 10.1371/journal.pgen.0030141 (PMC1950956; doi:10.1371/journal.pgen.0030141)
Supplement: Supplementary file 14 [file pgen.0030141.st002.doc]

**Table S2**

**Correlation of DSB hotspots and gene ontology (GO) classes**

| **GO term** | **P value for term-overrepresentation among all genes with adjacent Rec12 peak** |
| --- | --- |
| GO:0005886 Plasma membrane | 3.82 x 10^-12^ |
| GO:0044459 Plasma Membrane Part  (Inclusion of GO:0005886) | 1.37 x 10^-5^ |
| GO:0031224 Intrinsic to membrane | 8.6 x 10^-6^ |
| GO:0016021 Integral to Membrane  (Inclusion of GO:0031224) | 1.17 x 10^-5^ |
| GO:0044425 Membrane Part  (Inclusion of GO:0031224) | 8.63 x 10^-5^ |
| GO:0051704 Interaction between Organisms | 2.02 x 10^-6^ |
| GO:0000746 Conjugation  (Inclusion of GO:0051704) | 1.11 x 10^-5^ |
| GO:0019953 Sexual Reproduction  GO:0000747 Conjugation with Cellular  Fusion  (Inclusions of GO: 0000746) | 3.17 x 10^-5^ |
| GO:0005386 Carrier Activity | 2.23 x 10^-7^ |
| GO:0015291 Porter Activity  GO:0015290 Electrochemical Potential-  Driven Transporter Activity  (Inclusions of GO: 0005386) | 9.21 x 10^-7^ |
| GO:0003700 Transcription Factor Activity | 2.96 x 10^-7^ |

Results are from GOstat using GeneDB *S. pombe* background list. Only terms with p <0.0001 are shown.
