# Supplementary material for: A Discrete Class of Intergenic DNA Dictates Meiotic DNA Break Hotspots in Fission Yeast
Source: PLoS Genet. 2007 Aug 24;3(8):e141. doi: 10.1371/journal.pgen.0030141 (PMC1950956; doi:10.1371/journal.pgen.0030141)
Supplement: Supplementary file 15 [file pgen.0030141.st003.doc]

**Table S3**

***S. pombe*** strains

| Strain number | Genotype**^a^** |
| --- | --- |
| GP1979 | *h^-^ ade6-M26/ade6-52 lys3-37/+ pro1-1/+ +/ura1-17 pat1-114/ pat1-114 end1-458/end1-458* |
| GP3087 | *h^-^smt0/h^-^smt0 rad50S/rad50S pat1-114/pat1-114 ade6-M26/ade6-M26 end1-458/end1-458 lys4-95/+ his4-239/+* |
| GP3121 | *h^+^/h^-^smt0 +/rad50S ade6-52/ade6-M210 +/ura4-D18* |
| GP3124 | *h^+^/h^-^smt0 rad50S/rad50S ade6-52/ade6-M210 +/ura4-D18* |
| GP3718 | *h^+^ ade6-3049 rad50S pat1-114 end1-458* |
| GP4999 | *h^-^ ade6-3049 ura4-D18* |
| GP5387 | *h^+^ ade6-M26 ura4-D18 rec12-169::kanMX6* |
| GP5411 | *h^+^ rad50S pat1-114* |
| GP5622 | *h^-^ rec12-171::ura4^+^ ura4-D18 ade6-3049* |
| GP5960 | *h^-^ rec12-201::6His-2FLAG ura4-D18 ade6-3049* |
| GP5961 | *h^-^ rec12-202::6His-2HA ura4-D18 ade6-3049* |
| GP6013 | *h^+^ rec12-201::6His-2FLAG pat1-114 rad50S ade6-3049* |
| GP6203 | *h^-^/h^-^ rec12-201::6His-2FLAG/12-201::6His-2FLAG pat1-114/ pat1-114 rad50S/rad50S ade6-3049/ ade6-3049 lys4-95/+ his4-239/+* |
| GP6204 | *h^+^/h^+^ rec12-202::6His-2HA/rec12-202::6His-2HA pat1-114/pat1-114 rad50S/rad50S ade6-3057/ade6-3057 lys4-95/+ his4-239/+* |
| GP6233 | *h^-^/h^-^ rec12-201::6His-2FLAG/12-201::6His-2FLAG rec10-175::kanMX6/rec10-175::kanMX6 pat1-114/pat1-114 rad50S/rad50S ade6-3049/ade6-3049 lys4-95/+ his4-239/+* |

**^a^** Alleles other than mating-type and auxotrophies are described in the following references: *ade6-3049* (Steiner and Smith, 2005), *ade6-M26* (Schuchert et *al.*, 1991), *rec12-169::kanMX6* (Davis and Smith, 2003), *rec12-171::ura4^+^* (Davis and Smith, 2003), *pat1-114* (Iino and Yamamoto, 1985), *rad50S* (Farah et *a*l., 2002), and *rec10-175::kanMX6* (Ellermeier and Smith, 2005). The *ade6-3049* allele is a derivative of the *ade6-3011* DSB and recombination hotspot allele (Steiner et al., 2002), with an extraneous second mutation removed (Steiner and Smith, 2005), and shows the same level of DSB formation (~11%) as that with *ade6-3011* (Steiner et al., 2002). Genealogies are available upon request.
