# Supplementary material for: A Discrete Class of Intergenic DNA Dictates Meiotic DNA Break Hotspots in Fission Yeast
Source: PLoS Genet. 2007 Aug 24;3(8):e141. doi: 10.1371/journal.pgen.0030141 (PMC1950956; doi:10.1371/journal.pgen.0030141)
Supplement: Supplementary file 16 [file pgen.0030141.st004.doc]

**Table S4**

Primers for plasmid and allele construction and PCR analysis

| Primer | Nucleotide sequence | Use |
| --- | --- | --- |
| oYL1 | 5’ CAATTTTAAAGAGAATTCTTGG 3’ | *rec12* cDNA |
| oYL2 | 5’ AGTAAAAAAAATGAATTCGAATG 3’ | *rec12* cDNA |
| oJF08 | 5' CGGGATCCCATAGTTGTTTAGGATTTCAAATTTCGATC 3' | pJF17 |
| oJF09 | 5' CGGGATCCCTGTAATTATTTTGATTAGATGGTGTAGATTTATC 3' | pJF17 |
| oJF12 | 5’GTGGAAAGCACTCAGTACGTcatcatcaccatcaccatACGCGTTAAGTTCTTGGATAGTTCAACCTAC 3’ | 6His tag and pJF19 |
| oJF21 | 5’ CGCGAGATTATAAAGATGACGATGACAAGGACTACAAGGATGATGACGATAAGA 3’ | 2FLAG tag and pJF31 |
| oJF22 | 5’ CGCGTCTTATCGTCATCATCCTTGTAGTCCTTGTCATCGTCATCTTTATAATCT 3’ | 2FLAG tag and pJF31 |
| oJF27 | 5’ CGCGATATCCTTATGATGTTCCAGATTACGCTTATCCATACGATGTTCCT  GATTATGCTA 3’ | 2HA tag and pJF35 |
| oJF28 | 5’ CGCGTAGCATAATCAGGAACATCGTATGGATAAGCGTAATCTGGAACATC  ATAAGGATAT 3’ | 2HA tag and pJF35 |
| OL1647 | 5’ cggtgcaactttgcatctttatgg 3’ | PCR at *ade6* |
| OL1646 | 5’ aattcatctaaaatgacggcagc 3’ | PCR at *ade6* |
| OL1381 | 5’ cgagaccaagaggaatgc 3’ | PCR at *mbs1* |
| OL1382 | 5’ gcccccctgacttttccc 3’ | PCR at *mbs1* |
| OL1692 | 5’ gcacaagtcaatcacacc 3’ | PCR at *ura1* |
| OL1032 | 5’ gcgcatcctgatattagc 3’ | PCR at *ura1* |
